# Electron‐Deficient Organic Molecules Based on B←N Unit: A N‐Type Room‐Temperature Chemiresistive Sensors with Moisture Resistance

**DOI:** 10.1002/advs.202409890

**Published:** 2024-11-14

**Authors:** Binbin Wang, Yali Xing, Kewei Zhang, Zhong Wang, Yanzhi Xia, Xiaojing Long

**Affiliations:** ^1^ State Key Laboratory of Bio‐fibers and Eco‐textiles Collaborative Innovation Center of Shandong Marine Biobased Fibers and Ecological Textiles Institute of Marine Biobased Materials College of Materials Science and Engineering Qingdao University Qingdao 266071 P. R. China; ^2^ Qingdao Institute of Bioenergy and Bioprocess Technology Chinese Academy of Sciences Qingdao 266101 P. R. China

**Keywords:** ammonia sensing, electron‐deficient B←N unit, extended A‐D‐π‐D‐A, modulated energy bandgap, n‐type organic small molecules

## Abstract

Organic molecules with tailorable chemical structures, high stability, and solution processability have great potential in the sensing field. Compared with p‐type organic small molecules (OSMs), the electron‐dominated n‐type analogs show superior conductivity when exposed to reducing gases, which can achieve outstanding sensor signal‐to‐noise ratios. However, inadequate humidity resistance at room temperature hinders the development of such molecules. Herein, an A‐D‐π‐D‐A molecular design strategy is proposed based on electron‐deficient B←N units, which results in effective intramolecular charge transport and sensitive responses by extending the π‐conjugation bridge. As a result, the ST‐2BP with A‐D‐π‐D‐A configuration shows a prominent sensitivity of 787 (R_a_/R_g_) in 20 ppm NH_3_ at room temperature and an almost initial and stable response under different relative humidity conditions, which is the highest among currently reported OSM sensors. Supported by theoretical calculations and in situ FTIR spectra, it is revealed that B←N units, which function as the active centers mediate the specific ammonia adsorption. This study provides a new understanding of the design of high‐performance room temperature gas sensing materials by decorating B←N units.

## Graphical abstract

An extended A‐D‐π‐D‐A molecular design strategy is reported to regulate the energy level and bandgap of organic small molecules NH_3_ sensing materials by introducing stable boron dipyrromethene and π‐extended stilbene. The extended A‐D‐π‐D‐A type **ST‐2BP** organic small molecule with low lowest unoccupied molecular orbital (LUMO) energy and high electron cloud density exhibits a high specificity response to NH_3_, with the electron‐deficient B←N units as active sites.

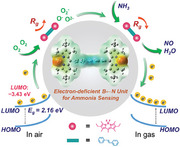

## 1 Introduction

**Scheme 1:**
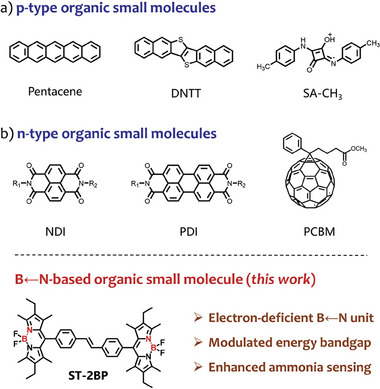
Examples of p‐type organic small molecules, n‐type organic small molecules, and B←N‐based n‐type organic small molecules (This work).

Organic semiconductors, renowned for their versatility in tuning chemical structures, solubility, and facile fabrication, have emerged as pivotal elements in developing innovative electrochemical sensing platforms.^[^ ^1^ ^]^ Capitalizing on their charge transfer capabilities and intermolecular interactions, efficient p‐type and n‐type organic semiconductor sensors demonstrate the potential to achieve precise and efficient responses to specific external chemical stimuli, notably including hazardous gases such as ammonia. Specifically, p‐type organic small molecules (OSMs), including pentacene, DNTT, and SA‐CH_3_, exhibit hole‐dominated charge transport properties, conferring exceptional environmental resilience and proven efficacy in detecting oxidizing gases such as NO_2_, NO, and Cl_2_.^[^ ^2^ ^]^ Nevertheless, their conductivity undergoes a notable decline upon exposure to reducing gases (NH_3_, H_2_S, CO), compromising the signal‐to‐noise ratio and thereby hindering widespread practical implementation (**Scheme** **1**).^[^ ^3^ ^]^ In stark contrast, n‐type organic molecules, characterized by inherent electron deficiency and low lowest unoccupied molecular orbital (LUMO) energies, demonstrate pronounced electron transfer capabilities.^[^ ^4^ ^]^ This unique attribute renders them particularly adept at interacting with NH_3_, which possesses lone pair electrons, leading to an enhanced conductivity upon contact with reducing gases.^[^ ^5^ ^]^ This conductivity augmentation presents a distinct advantage in the realm of sensing and detection, offering promising applications involving reducing gas analytes.^[^ ^6^ ^]^

Traditionally, high‐performance n‐type OSMs have a planar rigid molecular structure across the entire extended π‐electron framework, which promotes carrier transport through intermolecular π–π overlap.^[^ ^7^ ^]^ The common structures are mainly dominated by naphthalene diimide (NDI), perylene diimide (PDI), and fullerene (PCBM).^[^ ^8^ ^]^ Their intermolecular weak dispersion or induction forces (<0.2 eV) can achieve gas sensing at room temperature, but it also leads to weak humidity resistance and recovery ability.^[^ ^9^ ^]^ The oxygen on the diimide unit easily forms hydrogen bonds with water, affecting the adsorption and desorption of ammonia, which limits the application of ammonia sensing in extreme environments. It is difficult to modify or functionalize the structure core of the diimide group further effectively.^[^ ^10^ ^]^ To expand the scope of organic sensors and advance the development of high‐performance NH_3_ sensing materials, a new concept of designing n‐type building blocks needs to be developed.

Boron dipyrromethene (BODIPY), a commonly used B←N‐containing organoboron compound, contains a six‐membered heterocycle and two pyrrole rings connected by a methionine bridge bond, which have extremely high environmental stability and are almost unaffected by acidity and alkalinity.^[^ ^11^ ^]^ In addition, its molecular skeleton is inherently uncharged as a whole,^[^ ^12^ ^]^ avoiding the possibility of electrostatic interaction with polar molecules (H_2_O) in the air, which effectively addresses relatively slow and delayed detection caused by the humidity resistance of n‐type molecules at room temperature. More importantly, electron‐deficient B←N units have a good affinity for ammonia molecules with lone pair electrons, and can further adjust the intramolecular electron density.^[^ ^13^ ^]^ Therefore, maintaining accurate molecular structure as well as efficiently optimizing the charge distribution using electron‐deficient B←N building block strategy can achieve significant progress in sensing performance.

Herein, we synthesized a series of n‐type OSMs (**ST‐2BP** and **ST‐BP**) by combining π‐extended stilbene with BODIPY based on electron‐deficient B←N units. The as‐prepared extended A‐D‐π‐D‐A type **ST‐2BP** exhibits excellent sensitivity, recoverability, and selectivity, which is one of the highest performances among all OSMs. More donor–acceptor (D–A) modification can favor forward intramolecular charge separation, and the introduction of π‐extended units further suppresses backward charge recombination, which enhances the electron‐deficient properties of B←N units. The inherent low LUMO energy and narrow bandgap of **ST‐2BP** help to capture Lewis alkali gases and accelerate the reaction conversion process, which displays a more prominent NH_3_ sensing response than that of single BODIPY (BP), stilbene (ST), or the A‐D‐π‐D type **ST‐BP** at room temperature. Furthermore, the fabricated **ST‐2BP** sensor maintains an almost initial and stable response under different relative humidity conditions. Theoretical calculations and in situ, FT‐IR spectroscopy confirmed the charge transfer from NH_3_ to electron‐deficient B←N units during the sensing process. This strategy to decorate electron‐deficient B←N units into n‐type organic sensing materials in a controllable manner, and understand the charge distribution and sensing mechanism provides a new approach for the design of high‐performance electrochemical sensing materials.

## 2 Results and Discussion

The chemical structures of the BP‐based molecules are illustrated in Figure S1 (Supporting Information). **ST‐2BP** and **ST‐BP** were synthesized via acyl chlorination and DDQ oxidation methods, respectively. The molecular structure was characterized by ^1^H NMR, ^11^B NMR, ^13^C NMR, mass spectrum, and elemental analysis. Positive ion MALDI‐TOF mass spectra of **ST‐2BP**/**ST‐BP** (Figure S2, Supporting Information) displayed the m/z value of ions (784.6/482.4) that is comparable to the theoretical molecular weights. The materials with stable BP‐based structures show excellent thermal stability with a thermal decomposition temperature (*T*_d_, 10% weight loss) higher than 255 °C (Figure S3, Supporting Information), which provides a basis for achieving stable room temperature sensing.

**Figure 1:**
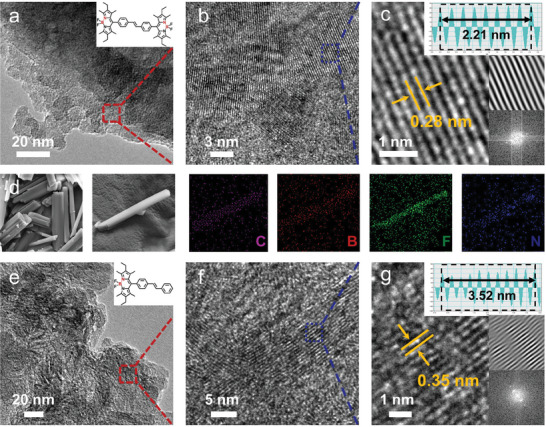
Various magnification TEM images of a−c) **ST‐2BP** and e−g) **ST‐BP** (insets: fast Fourier transform patterns of the corresponding images, the lattice fringe image presented by Fourier transform, and the corresponding lattice spacing profile). d) SEM images and EDS mapping of **ST‐2BP**.

Scanning electron microscopy (SEM) images revealed a uniform rod‐shaped structure of **ST‐2BP** and **ST‐BP** (Figure S4, Supporting Information), which can be well deposited on the interdigital electrode to form a dense network pattern. The extended π‐conjugated skeleton enhances the stacking of molecules and improves their charge transfer performance. Energy dispersive X‐ray spectroscopy (EDS) images further confirmed the uniform distribution of C, B, F, and N elements (**Figure** 1d) and excluded the influence of residual DDQ oxidants. Furthermore, the lattice fringe was observed by high‐resolution transmission electron microscopy (HRTEM) with a d‐spacing of 0.28 nm for **ST‐2BP** (Figure 1a−c) and 0.35 nm for **ST‐BP** (Figure 1e−g). High crystallinity is conducive to excellent charge separation capabilities, which promotes efficient sensing. To further evaluate the electronic states of materials, X‐ray photoelectron spectroscopy (XPS) was employed to investigate bonding states (Figure S5, Supporting Information). The N 1s spectrum can be fitted into two peaks, respectively attributed to pyrrole‐N (C═N─C) (399.5 eV for **ST‐2BP** and 399.4 eV for **ST‐BP**) and B─N bonds (401.8 eV for **ST‐2BP** and 401.6 eV for **ST‐BP**). Interestingly, compared to **ST‐BP**, the characteristic N 1s and B 1s peaks of **ST‐2BP** are shifted to higher binding energy, which is due to the different distribution of electron‐deficient B←N units. The infrared spectrum showed the alkyl characteristic peak of the BP‐based molecules at 2863−2958 cm^−1^, as well as the carbon characteristic peak of the styrene para position for **ST‐BP** at 1604 cm^−1^ (Figure S6, Supporting Information). UV–vis and fluorescence spectra of **ST‐2BP**, **ST‐BP**, and ST were measured in 10^−6^ m CH_2_Cl_2_ (Figure S7, Supporting Information). In addition, the degree of stable radical anion formation (SRAF) in solution is examined by observing the change of UV–vis spectra in the presence of n‐hexylamine (ammonia‐mimic).^[^ ^14^ ^]^ The extended A‐D‐π‐D‐A type **ST‐2BP** showed obvious SRAF behavior as a gradual increase of absorption band in the range of 250−330 nm. SRAF‐based materials demonstrated a significant increase in electrical conductivity by adsorption of Lewis alkaline gas (NH_3_).

**Figure 2:**
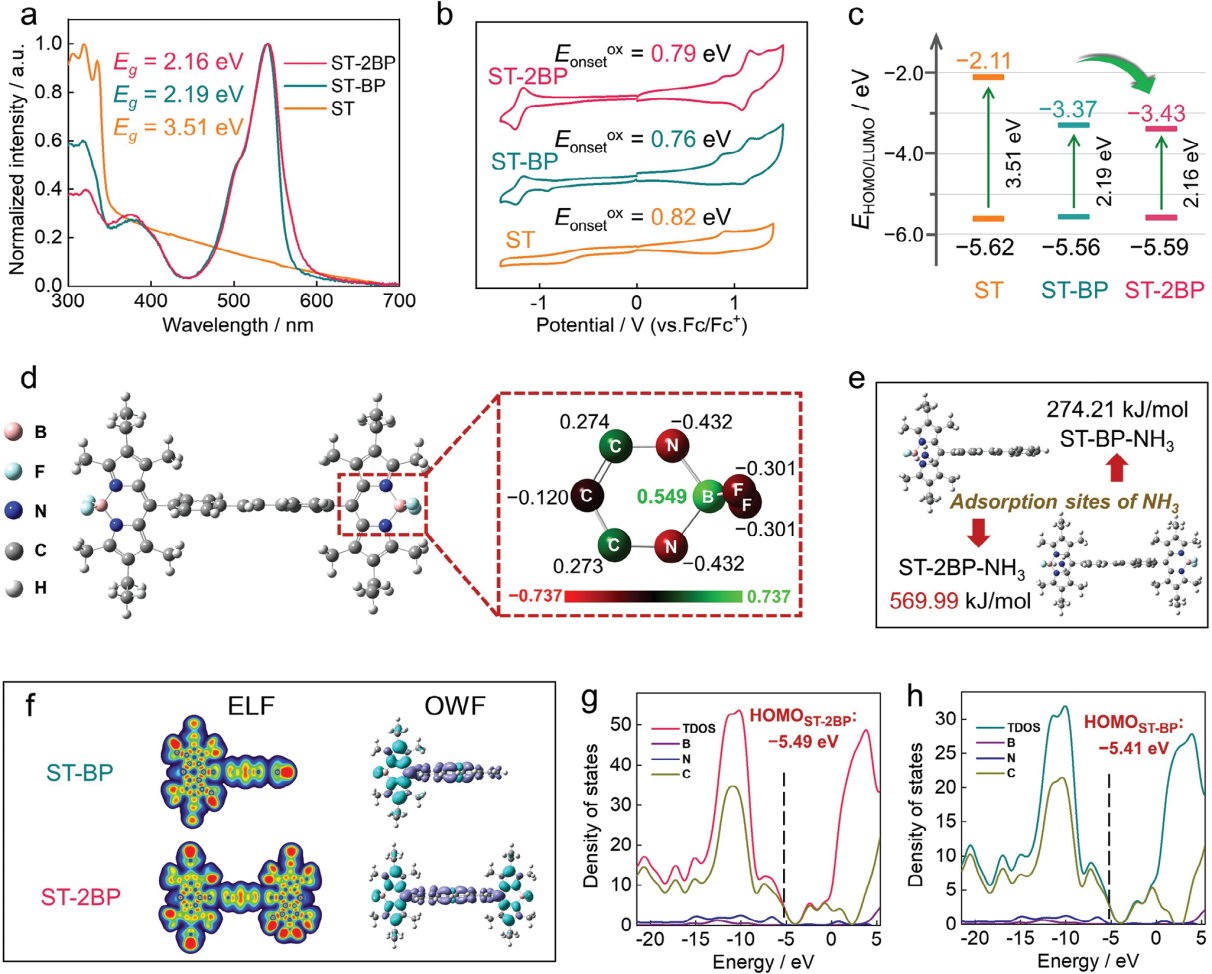
a−c) UV–vis DRS, cyclic voltammetric curves, and schematic representation of the LUMO/HOMO energy alignments of **ST‐2BP**, **ST‐BP**, and ST. d) Optimized molecular structure and the Mulliken charges on main atoms. e) Calculated adsorption energy of **ST‐2BP** and **ST‐BP** for NH_3_. f) Electron localization function (Left) and Orbit weight Fukui function (Right, Blue, and purple represent the positive and negative parts, respectively). The total density of states (TDOSs, purple lines), the partitional density of states (PDOSs) of C (green lines) and O (pink lines) for g) **ST‐2BP** and h) **ST‐BP**.

Constructing low LUMO energies to enhance the capture electrons ability for organic materials is usually an effective method to improve n‐type performance.^[^ ^15^ ^]^ The band structure of BP‐based film materials was evaluated using UV–vis diffuse reflectance spectra (DRS) (**Figure** **2**a; Figure S8, Supporting Information). The extended A‐D‐π‐D‐A type **ST‐2BP** was endowed with a narrow bandgap of 2.16 eV, requiring less energy for electron excitation.

Meanwhile, a cyclic voltammogram (CV) was characterized for calculating the energy level of these OSMs (Figure 2b). Based on bandgap and onset oxidation potential, the LUMO energy of **ST‐2BP** was calculated as −3.43 eV (Figure 2c). The low LUMO energies not only improve the electronic conductivity as an n‐type molecule but also enhance the ability to obtain electrons, which is beneficial for improving the detection effect of Lewis alkali gases. Density functional theory (DFT) calculations at B3LYP/6‐31G(d,p) level of theory were carried out to investigate the molecular configuration and charge distribution of BP‐based structure (Figure S9, Supporting Information). The molecular model shows delocalized highest occupied molecular orbitals (HOMO) and LUMO over the structure framework. In electrostatic potential surface maps, blue (positive charges) and red (negative charges) are concentrated in ST and BP parts (Figure S10, Supporting Information), respectively. The whole molecule exhibits regionalized charge distribution in **ST‐2BP** and **ST‐BP**.

Significantly, the D–A characteristic and extended π‐conjugated skeleton by the introduction of ST results in lower LUMO energies (**ST‐2BP** for −2.58 eV and **ST‐BP** for −2.49 eV) and narrower bandgaps (**ST‐2BP** for 2.91 eV and **ST‐BP** for 2.92 eV), which contribute to achieve stable NH_3_ sensing at room temperature.

To elucidate the NH_3_ adsorption sites of BP‐based molecules during the sensing process, we further calculated the optimized structure to explore the actual sensing reaction mechanism. The Millikan charge on the main atoms shows a high positive charge density of 0.549 at the B site (Figure 2d),^[^ ^16^ ^]^ which is beneficial for anchoring Lewis alkaline gases. We introduced the NH_3_ molecule into the initial molecular skeleton model and performed optimization calculations. Compared with other positions, the optimized model was more stable when NH_3_ was adsorbed to the B←N site (Figure 2e). The large adsorption energy of the extended A‐D‐π‐D‐A type **ST‐2BP** (569.99 kJ mol^−1^) indicates that NH_3_ has an effective affinity with the electron‐deficient B←N unit. The electron localization function (ELF) calculation of the model compounds further confirmed the active sites of the as‐prepared materials.^[^ ^17^ ^]^ The π‐extended **ST‐2BP** and **ST‐BP** enhanced the localization around the B←N unit region (Figure 2f), conducting the NH_3_ sensing process. Nucleophilic sites were further forecasted by the Fukui function to identify reaction sites. It is worth noting that the isosurface maps gradually increase from **ST‐BP** to **ST‐2BP**. The high distribution of the isosurface map in **ST‐2BP** will facilitate the adsorption of nucleophilic ammonia. Figure 2g,h shows the partitional density of states (PDOS) of the total, B atoms (purple line), N atoms (blue line), and C atoms (brown line) on **ST‐2BP** and **ST‐BP**. It can be observed that C plays a major role in the BP‐based molecules. The integral area of **ST‐2BP** is significantly larger, indicating that a stronger D–A characteristic by the introduction of more electron‐deficient B←N units enhances the electron density of the entire molecular skeleton. This enhancement can promote the interaction with NH_3_ during the sensing process.

**Figure 3:**
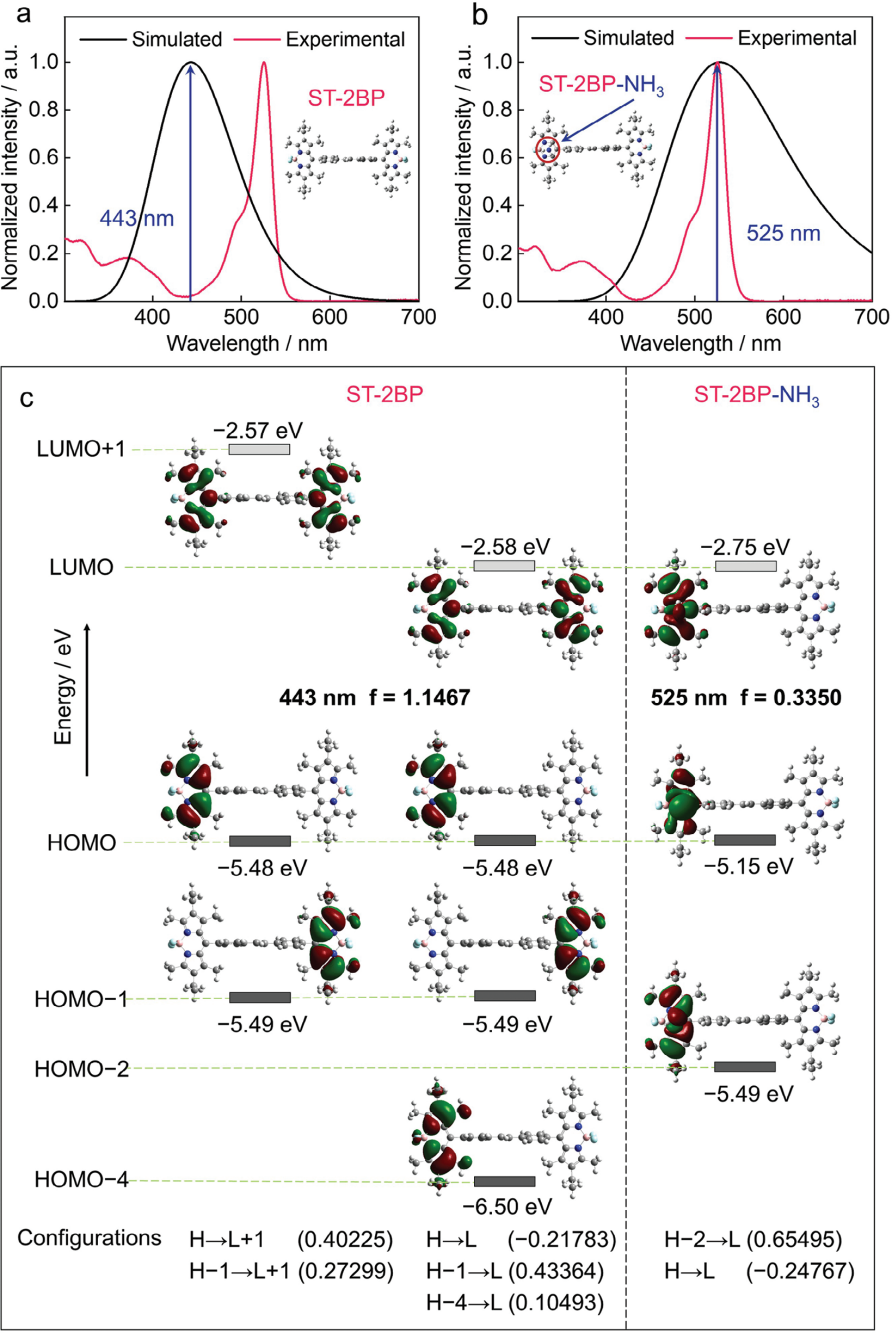
UV–vis spectra of a) **ST‐2BP** and b) **ST‐2BP‐NH_3_** obtained through theoretical calculation and testing. c) Kohn–Sham molecular orbitals of **ST‐2BP** and **ST‐2BP‐NH_3_**, together with the wavelengths and oscillator strengths for the S_0_→S_1_, S_0_→S_2_, S_0_→S_3_, and S_0_→S_5_ transitions, based on the DFT calculations.

To study the changes of NH_3_ adsorption on the sensing properties, we simulated UV–vis spectra of **ST‐2BP**, **ST‐2BP‐NH_3_**, **ST‐BP**, and **ST‐BP‐NH_3_** as well as calculated corresponding electronic transitions (**Figure** **3**a,b; Figure S11, Supporting Information).^[^ ^18^ ^]^ Figure 3c displays the electronic transitions and calculated molecular orbitals. The absorption maxima around 443 nm of the extended A‐D‐π‐D‐A type **ST‐2BP** is mainly assigned to a forbidden S_0_→S_2_ transition (HOMO→LUMO+1 and HOMO−1→LUMO) for **ST‐2BP**. After introducing NH_3_, the shoulder band of **ST‐2BP‐NH_3_** shifted red to 525 nm, and mainly attributed to a forbidden S_0_→S_3_ transition (HOMO−2→LUMO) for **ST‐2BP‐NH_3_**. It is worth noting that while the LUMOs of **ST‐2BP** are mainly distributed on the two symmetric BP cores, the LUMOs of **ST‐2BP‐NH_3_** are all localized on the BP core near NH_3_. Hence, the change in shoulder bands corresponds to the charge transfer from NH_3_ to **ST‐2BP**. Similarly, the shoulder band of around 436 and 366 nm of **ST‐BP‐NH_3_** attributed to allowed electronic transitions S_0_→S_2_ and S_0_→S_4_ (involving both HOMO−1→LUMO and HOMO−3→LUMO transitions) for **ST‐BP‐NH_3_**. The calculated spectrum wavelength is consistent with experimental data. These results reveal that NH_3_ significantly influences the electronic structures of these containing deficient‐electron B←N units, thus stimulating their efficient sensing properties.

**Figure 4:**
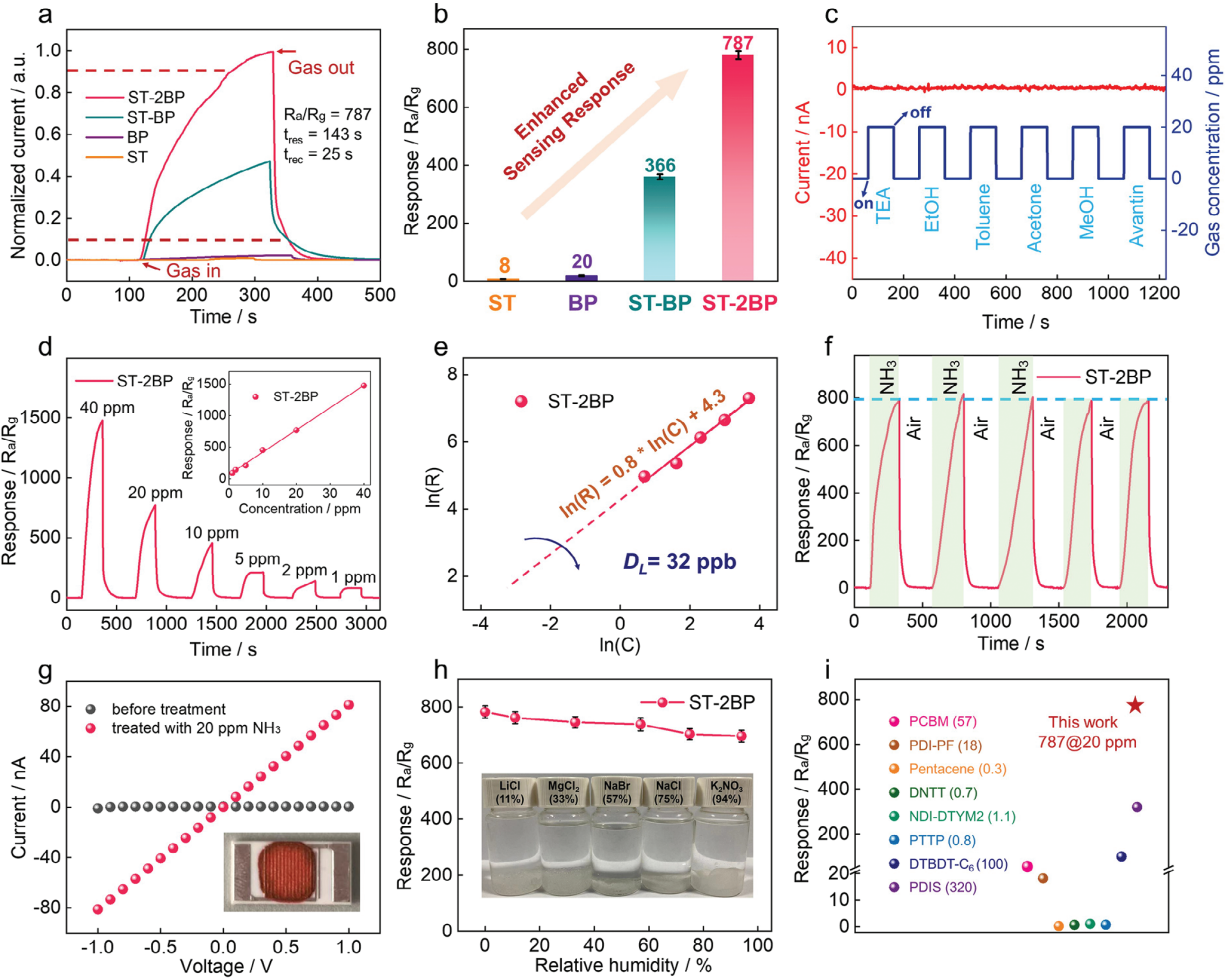
a) Normalized response–recovery time curves and b) sensing response values of **ST‐2BP**, **ST‐BP**, BP, and ST at room temperature (20 ppm NH_3_). c) Sensing current of **ST‐2BP** to 6 types of interfering gases (20 ppm). d) Sensing response curve of **ST‐2BP** to NH_3_ (1−40 ppm) (inset: The response value is linearly related to the NH_3_ concentration). e) Relationships between the sensor responses and NH_3_ concentrations. f) The cycling stability of the **ST‐2BP** sensor to 20 ppm NH_3_. g) The current (I)−voltage (V) curves. h) Response of the sensors to 20 ppm NH_3_ under different humidity levels (saturated salt solution for regulating relative humidity inset). i) The sensing response and corresponding NH_3_ concentration of reported OSMs and **ST‐2BP** (this work). (each experiment was independently tested three times; sample size n = 3; mean ± standard deviation (mean ± SD) was analyzed using Origin software; SD reflects the degree of dispersion among individual samples; a small SD means that the value of the test is close to the average; the P‐value indicates significant differences: **ST‐2BP** to BP: P < 0.0001; **ST‐BP** to BP: P < 0.0001; the statistical test was two‐sided testing, the α‐value was 0.05 and related P‐values were analyzed by a Student's two‐side test of GraphPad Prism software; P‐values less than 0.0001 indicate that the differences between **ST‐2BP** to BP and **ST‐BP** to BP is particularly significant).

We deposited these samples onto the interdigitated electrodes electrode with a gap of 0.15 mm and conducted thermal calcination to fabricate a stable gas sensor. NH_3_ sensing was tested at low or even room temperature (Figure S12, Supporting Information). Compared with pure ST or BP, the π‐extended **ST‐2BP** and **ST‐BP** with D–A characteristics reveal a much higher response to NH_3_ (**Figure** **4**a,b), demonstrating the advantage of D–A characteristic and extended π‐conjugated skeleton in improving the gas‐sensing behavior of OSMs. Further, the extended A‐D‐π‐D‐A type **ST‐2BP** with low LUMO energy exhibits a response value of 787 (R_a_/R_g_) at 20 ppm NH_3_, which is one of the highest performances among all OSMs. (Figure 4i; Table S1, Supporting Information). Under excellent charge transfer, the sensor delivers faster response and recovery time.^[^ ^19^ ^]^ In detail, the response time is 143 s and the recovery time is 25 s for **ST‐2BP**. As far as we know, this extremely fast recovery time has rarely been reported for NH_3_ sensing materials. By comparing the sensor response curves under different concentrations of NH_3_, the corresponding calibration curve demonstrates the linear relationship between sensor response and NH_3_ concentration (Figure 4d,e). The theoretical detection limit (*D_L_*) of **ST‐2BP** is calculated to be 32 ppb because *D_L_* is the value of gas concentration when the sensor response is three times greater than the standard deviation of the noise signal.

Selectivity is a vital criterion for evaluating the functionality of sensing materials. After being exposed to 6 common interfering gases and 4 irritating gases with a concentration of 20 ppm for 100 s, no significant current changes were observed, implying that **ST‐2BP** possessed excellent selectivity (Figure 4c; Figure S13). In addition, we evaluated the interaction between other gas and BP‐based molecules by conducting DFT calculations on the gas adsorption model (Figure S14, Supporting Information). It was worth noting that NH_3_ had the largest adsorption energy of 569.99 kJ mol^−1^ on the B←N unit portion of the extended A‐D‐π‐D‐A type **ST‐2BP**, which is higher than other gas molecules and supported the high selectivity of **ST‐2BP** toward NH_3_. In the presence of interfering gas (H_2_S, TEA, and NO_2_), the **ST‐2BP** sensor exhibits a similar response compared to the NH_3_ atmosphere, indicating its high anti‐interference performance (Figure S15, Supporting Information). Moreover, the NH_3_ sensing response of **ST‐2BP** showed minor changes during the cyclic test (Figure 4f), which results revealed that the extended A‐D‐π‐D‐A type **ST‐2BP** had outstanding cyclic stability, possibly caused by the stable π‐conjugated skeleton. The sensing test of the **ST‐2BP** sensor for seven weeks found that all reactions display initial response values, indicating excellent long‐term stability (Figure S16, Supporting Information). In addition, the ^1^H‐NMR test showed an identical structure of **ST‐2BP** after the cyclic stability test (Figure S17, Supporting Information). Notably, the conductivity of **ST‐2BP** has significantly improved under the NH_3_ atmosphere (Figure 4g). The sensor was tested using different saturated salt solutions to simulate relative air humidity (Figure 4h). The difference in NH_3_ response was not significant, evidencing sufficient humidity resistance. Furthermore, we tested the resistance response/recovery curve of **ST‐2BP** under 20 ppm NH_3_ (Figure S18, Supporting Information). The real‐time data measured by a multimeter shows that the ammonia released from spoiled seafood significantly reduces the resistance of the sensor (Figure S19, Supporting Information), which provides a possibility for detecting the freshness of seafood.^[^ ^20^ ^]^

**Figure 5:**
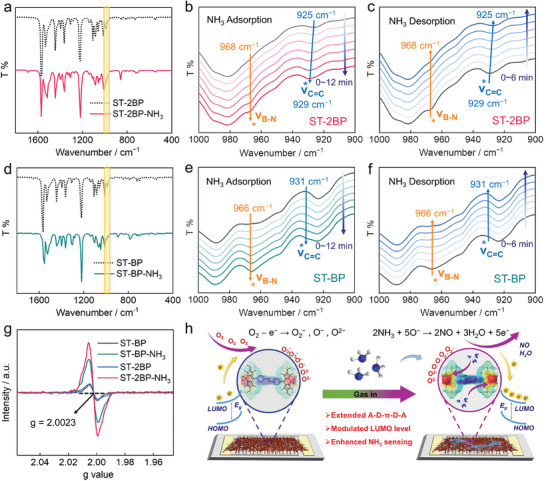
The FTIR spectra of a) **ST‐2BP** and d) **ST‐BP** before and after adsorption of NH_3_ in theoretical calculations. The in situ FTIR spectra of **ST‐2BP** after b) adsorption and c) desorption of NH_3_. The in situ FTIR spectra of **ST‐BP** after e) adsorption and f) desorption of NH_3_. g) EPR spectra of molecules before and after NH_3_ adsorption. h) Schematic diagram of NH_3_ adsorption and desorption process.

To understand the excellent sensing performance of BP‐based OSMs effectively, simulated infrared spectra and corresponding in situ FTIR spectra were obtained to prove possible interaction sites.^[^ ^21^ ^]^ Through the analysis of in situ FTIR spectra (**Figure** **5**b,e), two major vibrations of **ST‐2BP**/**ST‐BP** models correspond to the shift in B─N bending vibration (925/931 cm^−1^) and adjacent C═C bending vibration (968/966 cm^−1^), which is consistent with the simulation of theoretical calculation (Figure 5a,d), further proving the interaction between electron‐deficient B←N units and NH_3_. The desorption process is the opposite and can be completed in a faster time (Figure 5c,f), which is beneficial for rapid sensing recovery. It was worth noting that, compared to the A‐D‐π‐D type **ST‐BP**, a significant blueshift (925−929 cm^−1^) was observed in C═C bending vibration of the extended A‐D‐π‐D‐A type **ST‐2BP** after introducing NH_3_, which is attributed to powerful D–A characteristic changes the electronic state of the carbon skeleton, resulting in stronger interaction with ammonia gas. To investigate the effect of NH_3_ adsorption on the electronic structure of materials, we characterized the electron paramagnetic resonance (EPR) spectra in air and ammonia atmospheres (Figure 5g).^[^ ^22^ ^]^ All the tests show a Lorentzian line centered at a g value of ≈2.0023. Compared with the initial state, the EPR signals of **ST‐2BP** and **ST‐BP** under the NH_3_ atmosphere indicated a significant increase in free electrons after NH_3_ adsorption (Figure 5h), which helps to achieve a high response in the sensing field.

## 3 Conclusion

In summary, to overcome the lack of traditional n‐type organic molecules in structure design for chemiresistive gas sensing, we synthesized a series of electron‐deficient B←N‐based OSMs. The specifically extended A‐D‐π‐D‐A type **ST‐2BP**, synthesized from stilbene and boron dipyrromethene, has inherent low LUMO energies and narrow bandgap, which further regulates the electronic structure. This D‐A characteristic and the introduction of π‐extended units facilitate the separation and transport of molecular skeleton charges. Importantly, **ST‐2BP** exhibited highly specific and efficient sensing of 787 (R_a_/R_g_) in 20 ppm NH_3_ with one of the highest sensitivities among all reported organic small molecules. The fabricated extended A‐D‐π‐D‐A type **ST‐2BP** sensor maintains an almost initial and stable response under different relative humidity conditions. Notably, the sensing mechanism has been further explored by DFT calculations and in situ FTIR spectrum, which revealed the ammonia affinity of electron‐deficient B←N units as active sites and the electron transfer from NH_3_ to boron dipyrromethene. This work provides a new approach to designing organic electrochemical sensing materials via the electron‐deficient B←N construction strategy.

## Conflict of Interest

The authors declare no conflict of interest.

## Supporting information



Supporting Information

## Data Availability

The data that support the findings of this study are available in the supplementary material of this article.
