# Supplementary material for: Electron‐Deficient Organic Molecules Based on B←N Unit: A N‐Type Room‐Temperature Chemiresistive Sensors with Moisture Resistance
Source: Adv Sci (Weinh). 2024 Nov 14;11(48):2409890. doi: 10.1002/advs.202409890 (PMC11672309; doi:10.1002/advs.202409890)
Supplement: Supplementary file 1 — Supporting Information [file ADVS-11-2409890-s001.docx]

***Supporting Information for***

**Electron-Deficient Organic Molecules Based on B←N Unit: A n-Type Room-Temperature Chemiresistive Sensors with Moisture Resistance**

Binbin Wang, Yali Xing, Kewei Zhang, Zhong Wang, Yanzhi Xia, and Xiaojing Long*****

*To whom correspondence should be addressed. E-mail: Xiaojing Long ([longxj@qdu.edu.cn](mailto:longxj@qdu.edu.cn))

Supplementary Experimental Procedures

**Materials**

All reactions were performed under an argon atmosphere. Dichloromethane was dried before use. Other solvents and reagents were used without further purification unless otherwise mentioned.

1. **Synthesis of ST-2BP**

Starting materials of 4,4'-Stilbenedicarboxylic acid (521.3 mg, 1.864 mmol) were placed in a two-necked flask under argon, and then thionyl chloride (15 mL) and DMF (0.5 mL) was added. After the mixture was stirred at 80 °C for 12 h, the resulting precipitate was filtered, and washed with deionized water and dichloromethane. The solid was dried at 100 ℃ under vacuum for overnight to provide stilbene-4,4'-dicarbonyl dichloride (529.1 mg, 93.0 %).

Starting materials of stilbene-4,4'-dicarbonyl dichloride (201.4 mg, 0.659 mmol) and 2,4-Dimethyl-3-ethyl-1H-pyrrole (487.1 mg, 3.954 mmol) were placed in a two-necked flask under argon, and then dried dichloromethane (8 mL) was added. After the mixture was stirred at 50 °C for 18 h, boron trifluoride diethyl etherate (2.2 mL) and triethylamine (1.2 mL) were added, and the reaction continued at 50 °C for 2 hours. After cooling, extract with dichloromethane (120 mL) and wash the organic phase with water. After removing the solvents at reduced pressure, the product was purified by silica gel column chromatography (dichloromethane/hexane = 1/1). The **ST-2BP** was collected and dried in a vacuum overnight. Yield: 217 mg (42 %). ^1^H NMR (400 MHz, CDCl_3_, 25 °C): *δ* 7.69 (d, *J* = 8.0 Hz, 2H), 7.32 (d, *J* = 10.4 Hz, 3H), 2.54 (s, 6H), 2.32 (d, *J* = 7.6 Hz, 4H), 1.35 (s, 6H), 1.01**−**0.97 (m, 6H). ^11^B NMR (600 MHz, CDCl_3_) *δ* **−**0.14. ^13^C NMR (400 MHz, CDCl_3_, 25 °C): *δ* 153.80, 139.76, 138.26, 137.41, 135.36, 132.82, 130.71, 128.80, 127.13, 46.91, 29.67, 17.06, 14.60, 14.10, 12.50, 11.80, 8.65.

1. **Synthesis of ST-BP**

Starting materials of 4-formyl-trans-stilbene (137.7 mg, 0.662 mmol) and 2,4-Dimethyl-3-ethyl-1H-pyrrole (489.4 mg, 3.972 mmol) were placed in a two-necked flask under argon, and then dried dichloromethane (8 mL) and TFA (5 μL) was added. After the mixture was stirred in the dark at 50 °C for 18 h, DDQ (150.1 mg, 0.662 mmol) was added. After stirring in the dark at 50 °C for 2 h, boron trifluoride diethyl etherate (2.2 mL) and triethylamine (1.2 mL) were added, and the reaction continued at 50 °C for 2 hours. After cooling, extract with dichloromethane (120 mL) and wash the organic phase with water. After removing the solvents at reduced pressure, the product was purified by silica gel column chromatography (dichloromethane/hexane = 1/1). The **ST-BP** was collected and dried in a vacuum overnight. Yield: 121 mg (38 %). ^1^H NMR (400 MHz, CDCl_3_, 25 °C): *δ* 7.65 (d, *J* = 5.6 Hz, 2H), 7.56 (d, *J* = 4.8 Hz, 2H), 7.41**−**7.38 (m, 2H), 7.31**−**7.27 (m, 3H), 7.24**−**7.16 (m, 2H), 2.54 (s, 6H), 2.33**−**2.29 (m, 4H), 1.36 (s, 6H), 1.00**−**0.98 (m, 6H). ^11^B NMR (600 MHz, CDCl_3_) *δ* **−**0.14. ^13^C NMR (400 MHz, CDCl_3_, 25 °C): *δ* 153.70, 139.94, 138.33, 137.74, 136.95, 134.94, 132.74, 130.74, 129.64, 128.74, 128.68, 127.93, 127.81, 127.01, 126.57, 31.40, 30.16, 29.67, 17.05, 14.59, 12.48, 11.84.

1. **Synthesis of BP**

**BP** was synthesized from acetyl chloride and 2,4-dimethyl-3-ethyl-1H-pyrrole following the synthesis procedure of **ST-2BP**. Yield: 166 mg (82 %). ^1^H NMR (400 MHz, CDCl_3_, 25 °C): δ 2.60 (s, 3H), 2.50 (s, 6H), 2.42**−**2.38 (m, 4H), 2.33 (s, 6H), 1.05**−**1.03 (m, 6H).

**Characterization**

^1^H NMR and ^11^B NMR spectra were measured with a Bruker AV-400 spectrometer in CDCl_3_ at 25 °C. Chemical shift is reported in δ ppm using CDCl_3_ (7.26 ppm) for ^1^H NMR. Thermal analysis was performed on a TG 209F3 instrument under nitrogen flow at a heating rate of 10 °C min^–1^. UV-vis absorption spectra were measured with a Shimadzu UV-3600 spectrometer. Fluorescence spectra were measured with a Hitachi F-4500 spectrometer in spectral-grade solvents. The morphologies and structures of the samples were characterized by using field emission scanning electron microscopy (FESEM; JSM-7001F, JEOL, Tokyo, Japan) with an energy dispersive X-ray spectrometer (EDS) and the transmission electron microscopy (TEM; JEM-1011; JEOL Co., Japan) operated at an accelerating voltage of 100 kV. Cyclic voltammetry (CV) was performed on a CHI660a electrochemical workstation. The chemistry composition was analyzed by X-ray photoelectron spectroscopy (XPS) using an Axis Supra electron spectroscopy (KRATOS) with monochromatic 150 W Al Ka radiation. The current (I)-voltage (V) curves were measured by a source Meter (2612B, Keithley) at room temperature. The Solid-state UV-vis diffuse-reflectance spectra were recorded on a Cary 5000 UV-vis-NIR spectrophotometer using an integrated sphere accessory. Electron paramagnetic resonance (EPR) experiments were performed on a Bruker EMX PLUS spectrometer.

**Ammonia sensing measurement**

Sensing device fabrication and gas-sensing performance measurements: Dissolve 0.5 mg, 3.0 mg, and 6.0 mg of organic molecules in 100 µL dichloromethane, respectively, and coat 10 µL of the solution on the interdigitated electrodes (IDE) to form uniform sensing films of different thicknesses. After drying, the sensor components were aged overnight at 120 °C to improve stability.

Gas sensing measurements were carried out by employing a Keithley electrometer (2611B) integrated with a customized gas testing chamber (1.5 L) at an input signal of 3 V. The static liquid–gas distribution method was employed to calculate the volume (μL) toward the desired concentration (ppm) of test analytes. The concentration of test analytes (ammonia, acetone, triethylamine, methanol, and ethanol) was measured using eq (1) and injected into the evaporator inside the chamber.

$\boldsymbol{C}_{\boldsymbol{ppm}}\boldsymbol{=}\frac{\boldsymbol{\delta\times}\boldsymbol{V}_{\boldsymbol{r}}\boldsymbol{\times R\times T}}{\boldsymbol{M\times}\boldsymbol{P}_{\boldsymbol{b}}\boldsymbol{\times}\boldsymbol{V}_{\boldsymbol{b}}}\boldsymbol{\times}\boldsymbol{10}^{\boldsymbol{6}}$ eq (1)

where δ is the density of the analyte, Vr is the volume of analyte injected, R is the universal gas constant, T is the absolute temperature, M is the molecular weight, Pb is the pressure inside the chamber and Vb is the volume of the chamber. The required volume of test analytes was taken using a microliter syringe. The current of the sensor was continuously monitored until it reached the steady state in the presence of a desired concentration of test analytes. Once the steady state was reached, the chamber was exposed to the ambient atmosphere to ensure the reversibility of the sensor, and the response characteristics of the sensor were monitored continuously. The testing device and process are shown in the following figure:


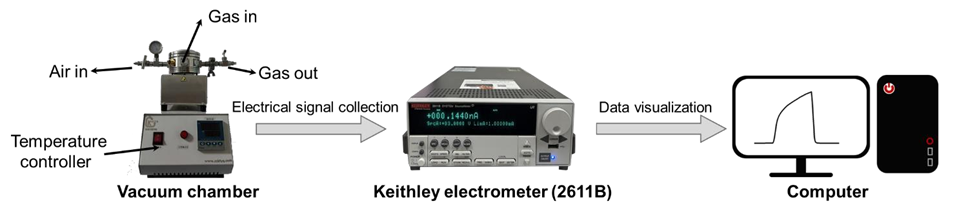


The response of the gas sensor (S) is defined as the ratio of the resistance (R_a_) of the sensor in the air to the resistance (R_g_) of the sensor after treatment with the measured gas, that is, S = R_a_/R_g_. According to the least-squares method of fitting in the linear regime, the theoretical detection limit (*D_L_*) of the gas sensor is the value of gas concentration when the sensor response is three times greater than the standard deviation of the noise signal (RMS_noise_), which can be derived as follow:

$$\boldsymbol{D}_{\boldsymbol{L}}\left( \boldsymbol{ppm} \right)\mathbf{=}\frac{\boldsymbol{3\cdot}\boldsymbol{rm}\boldsymbol{s}_{\boldsymbol{noise}}}{\boldsymbol{slope}}\mathbf{=}\frac{\mathbf{3}}{\boldsymbol{slope}}\boldsymbol{\cdot}\sqrt{\frac{\boldsymbol{V}_{\boldsymbol{x}^{\mathbf{2}}}}{\boldsymbol{N}}}$$

where N is the number of data points used in the plot fitting, V(x^2^) is the standard deviation of the data points used, and the slope is the slope of the fitting plot of response versus NH_3_ concentration.

**Transmission Electron Microscopy Testing**

Preparation of Transmission Electron Microscopy Samples: Take 1 mg of the previously prepared organic molecule (**ST-2BP**/**ST-BP**) solid powder and add 200 μL of ethanol. Disperse with ultrasound for 30 minutes, then add 3–5 drops of suspension onto a copper mesh. After the ethanol evaporates, perform TEM testing.

**Statistical Analysis**

Pre-processing of data: UV, CV, and other sensing test data were converted into TXT format by the corresponding instruments, and plotted by Origin software without normalization and evaluation of outliers. The data of SEM, TEM, and DFT calculations were in the form of pictures or numbers, which were directly drawn by PowerPoint software without conversion, normalization, and evaluation of outliers.

Data presentation: The sensing measurements were independently tested three times to avoid any incidental error. The related error bars (presented in the form of mean ± SD) were also shown in the manuscript and supporting information.

Sample size for each statistical analysis: The sample size of the related sensing measurements was three.

Statistical methods used to assess significant differences with sufficient details: The statistical test was two-sided testing, the α value was 0.05 and related P values were analyzed by Student’s two-side t test and showed in the manuscript and supporting information.

Software used for statistical analysis: The related software were Origin and PowerPoint.


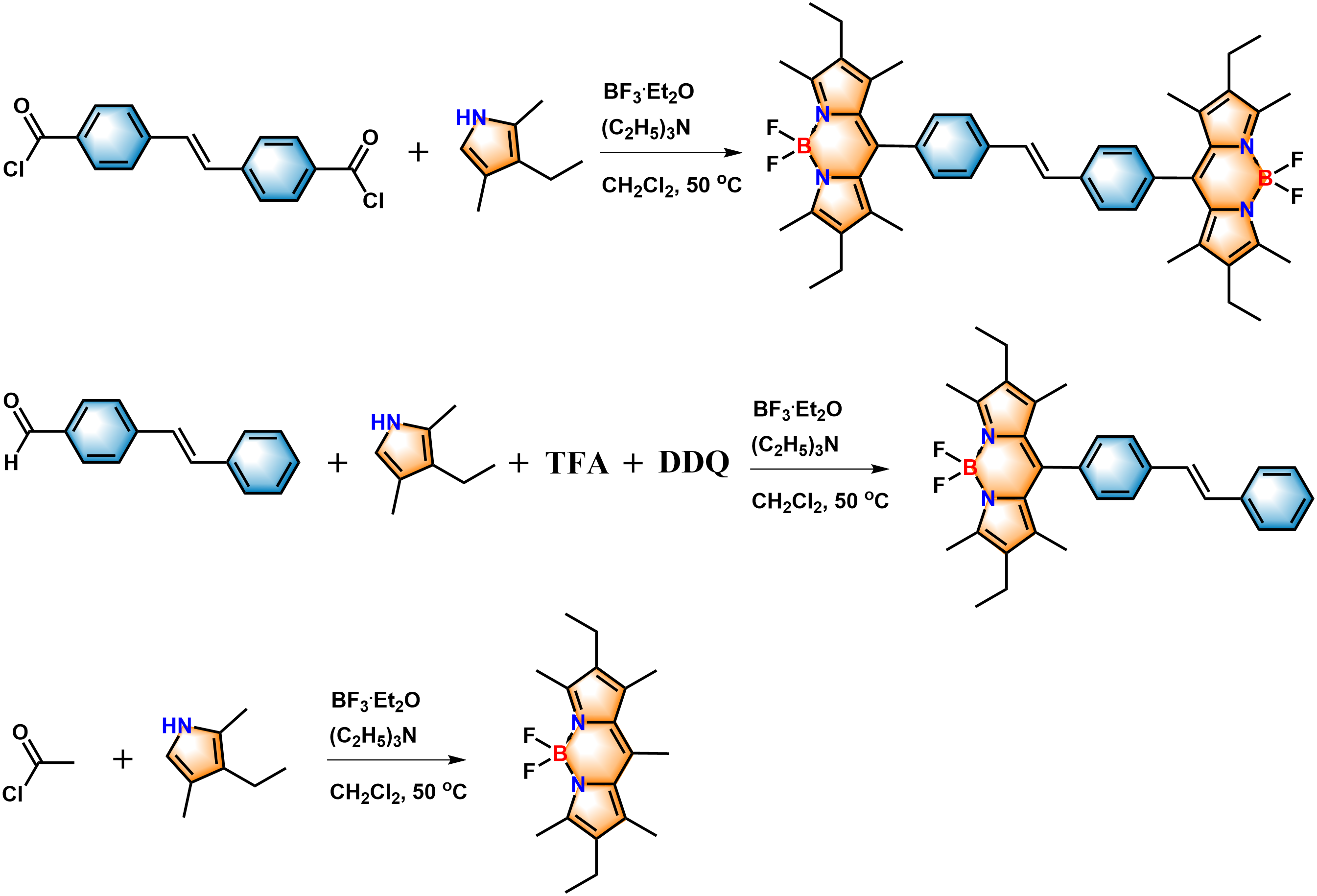


**Figure S1.** Synthesis method of **ST-2BP**, **ST-BP**, and BP.


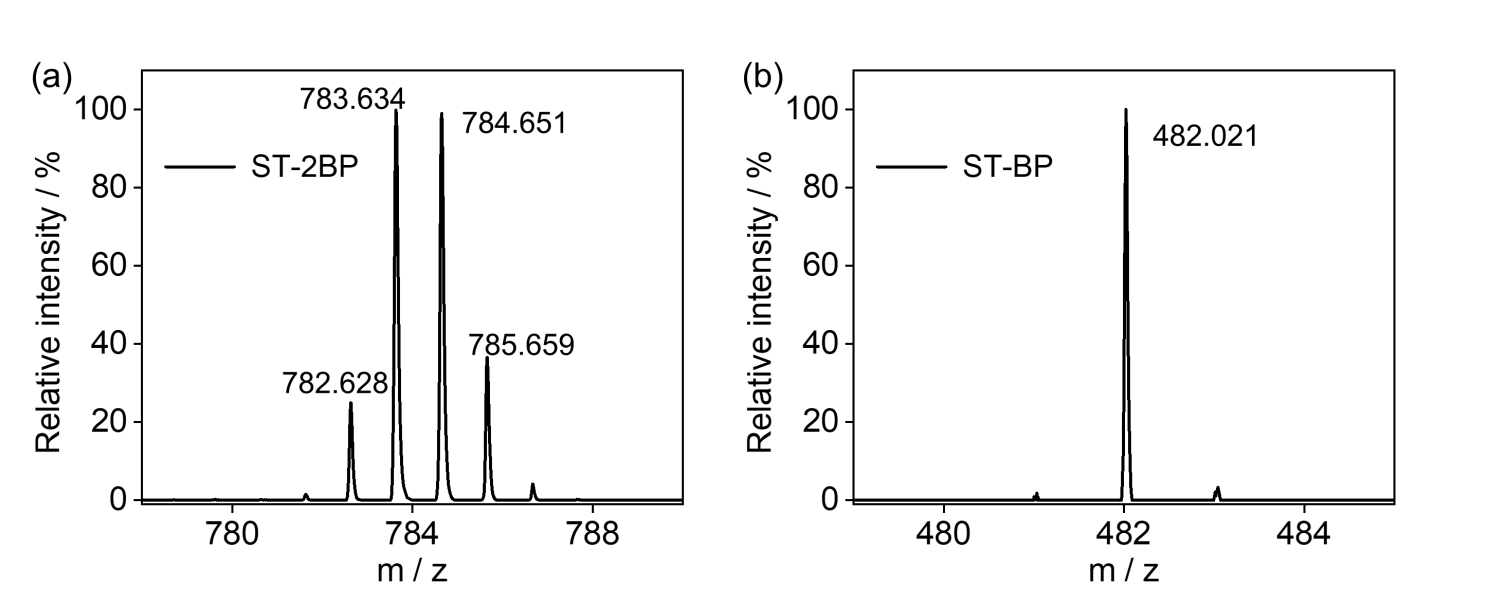


**Figure S2.** The mass spectra of (a) **ST-2BP** and (b) **ST-BP**.


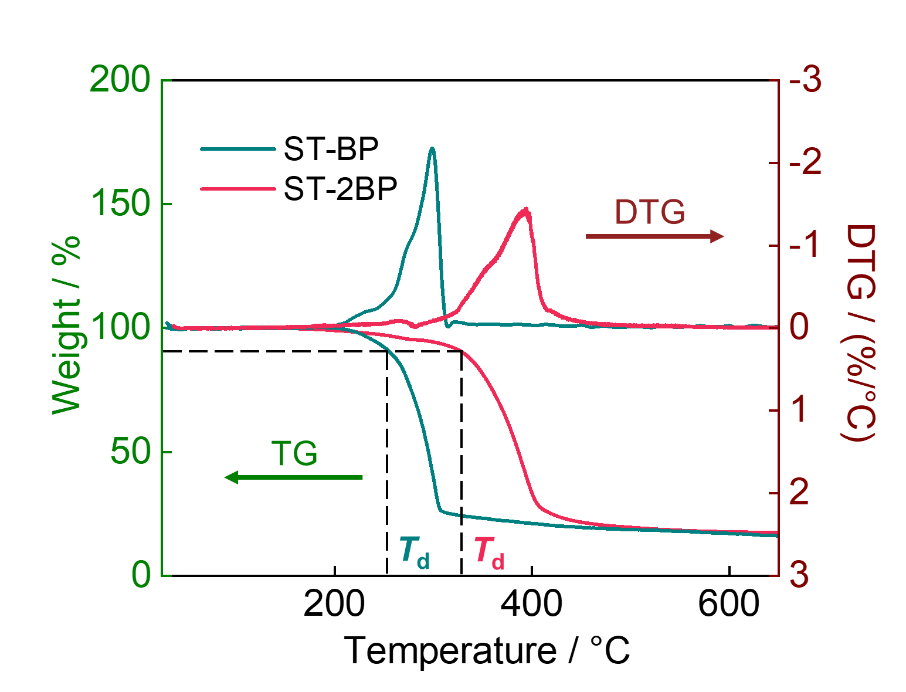


**Figure S3.** TGA and DTG curves of **ST-BP** and **ST-2BP**.


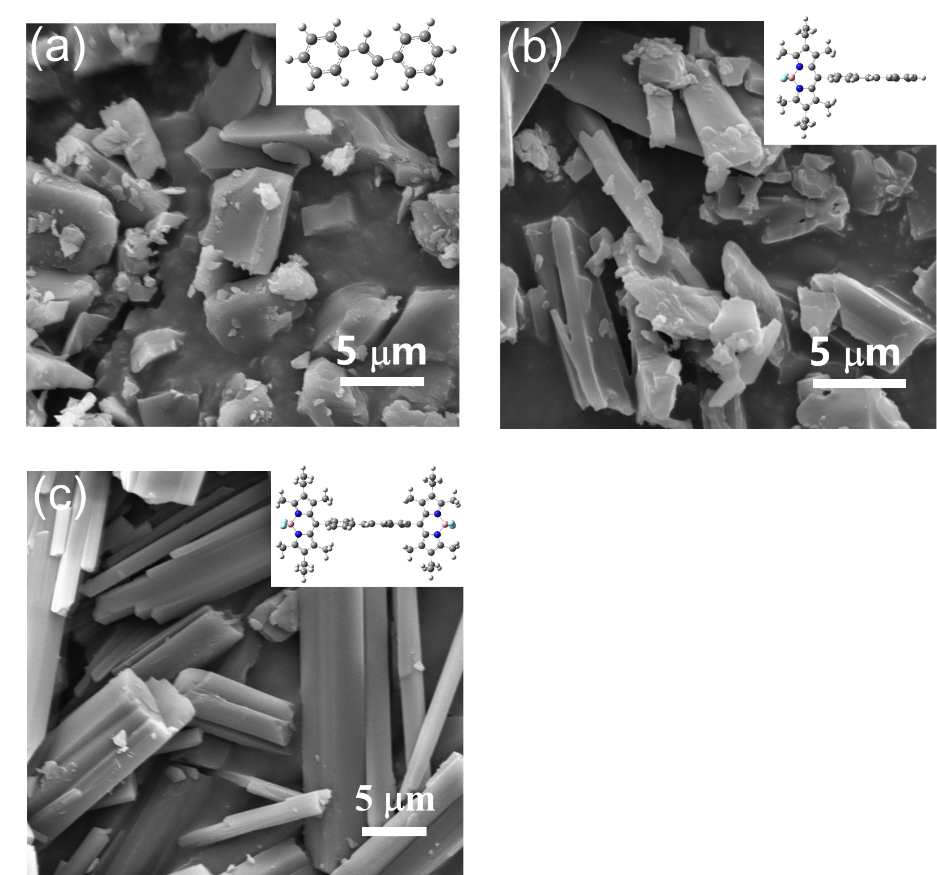


**Figure S4.** SEM images of (a) ST, (b) **ST-BP**, and (c) **ST-2BP**.


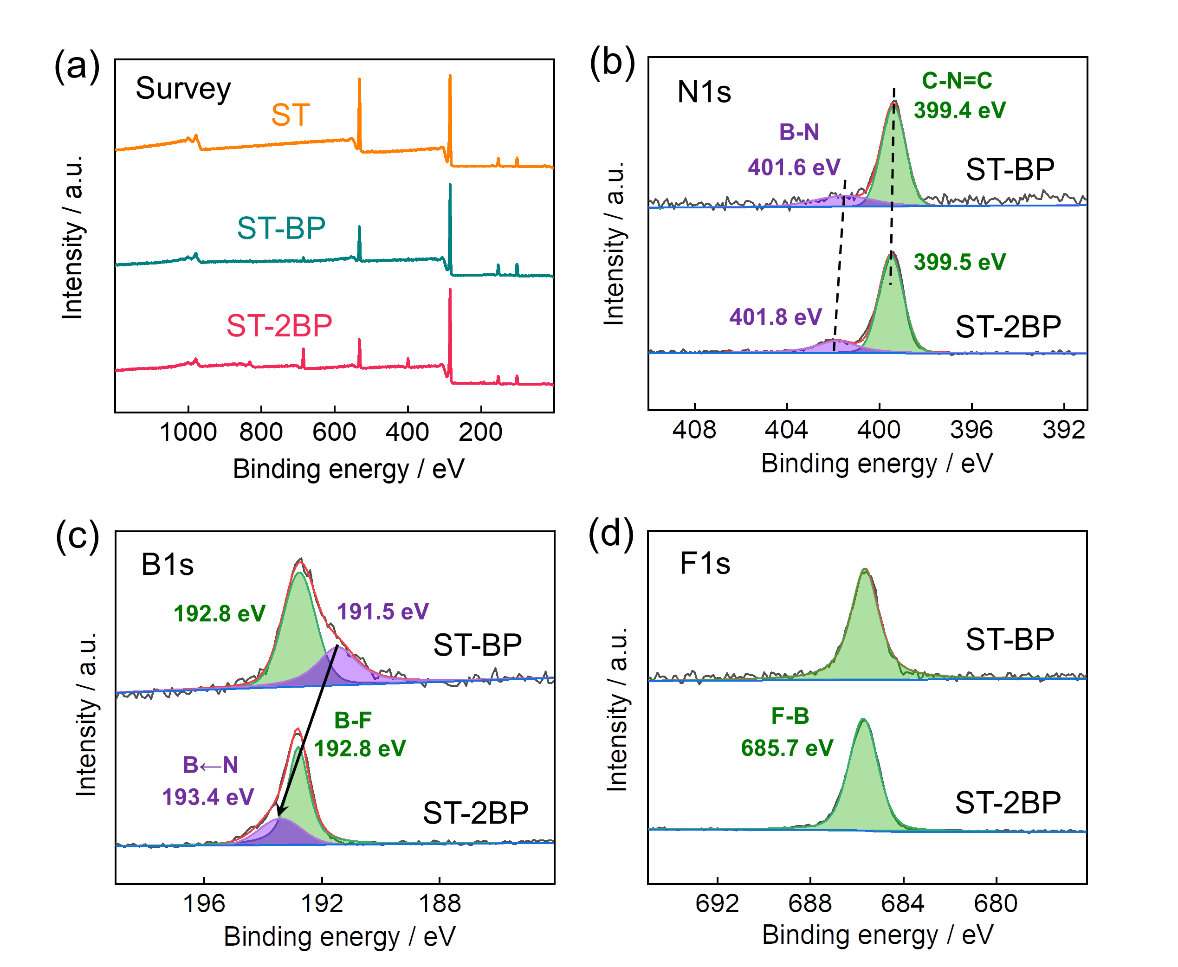


**Figure S5.** XPS spectra of (a) survey; (b) N 1s; (c) B 1s; (d) F 1s of ST, **ST-BP**, and **ST-2BP**.


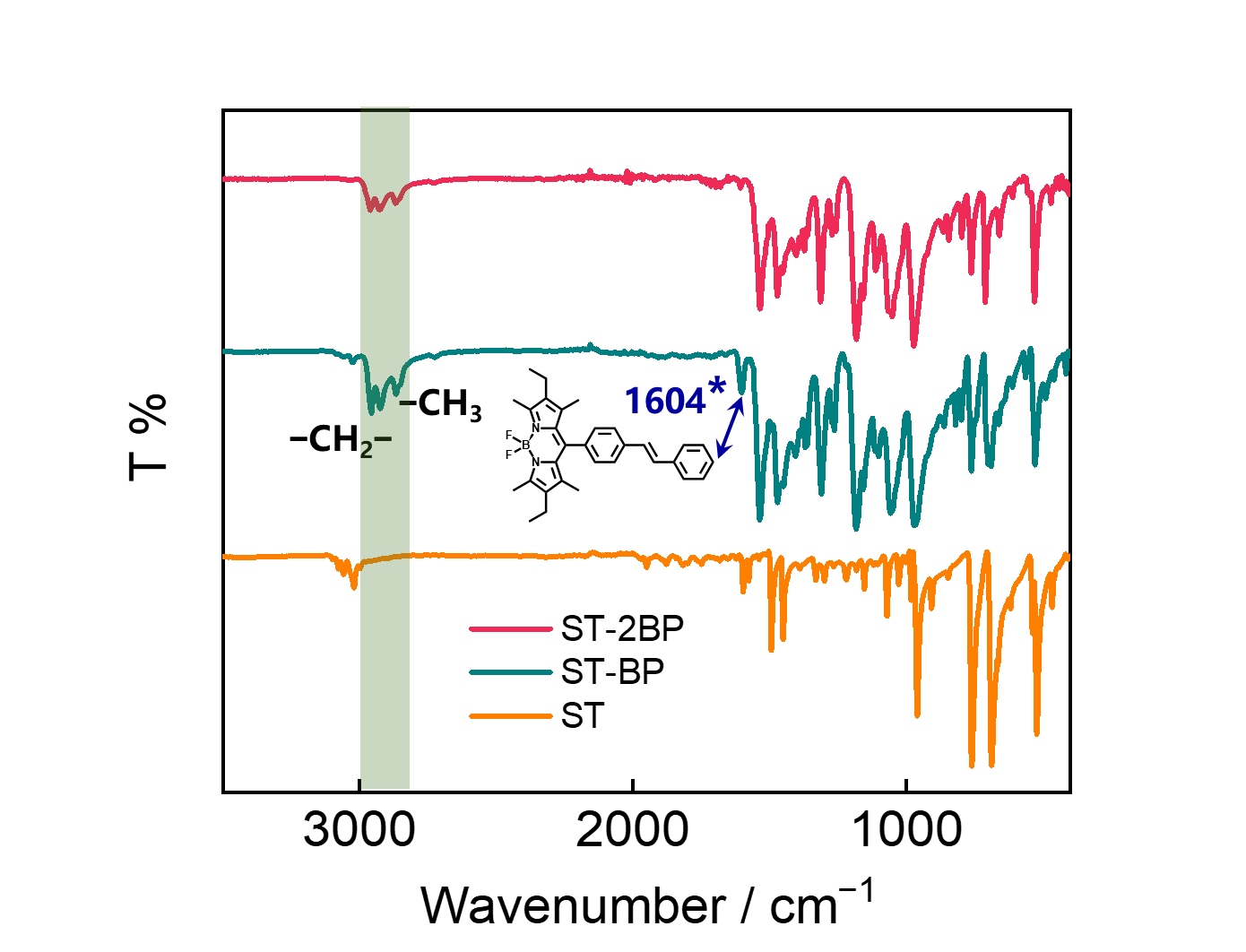


**Figure S6.** FT-IR spectra of **ST-2BP,** **ST-BP**, and ST.


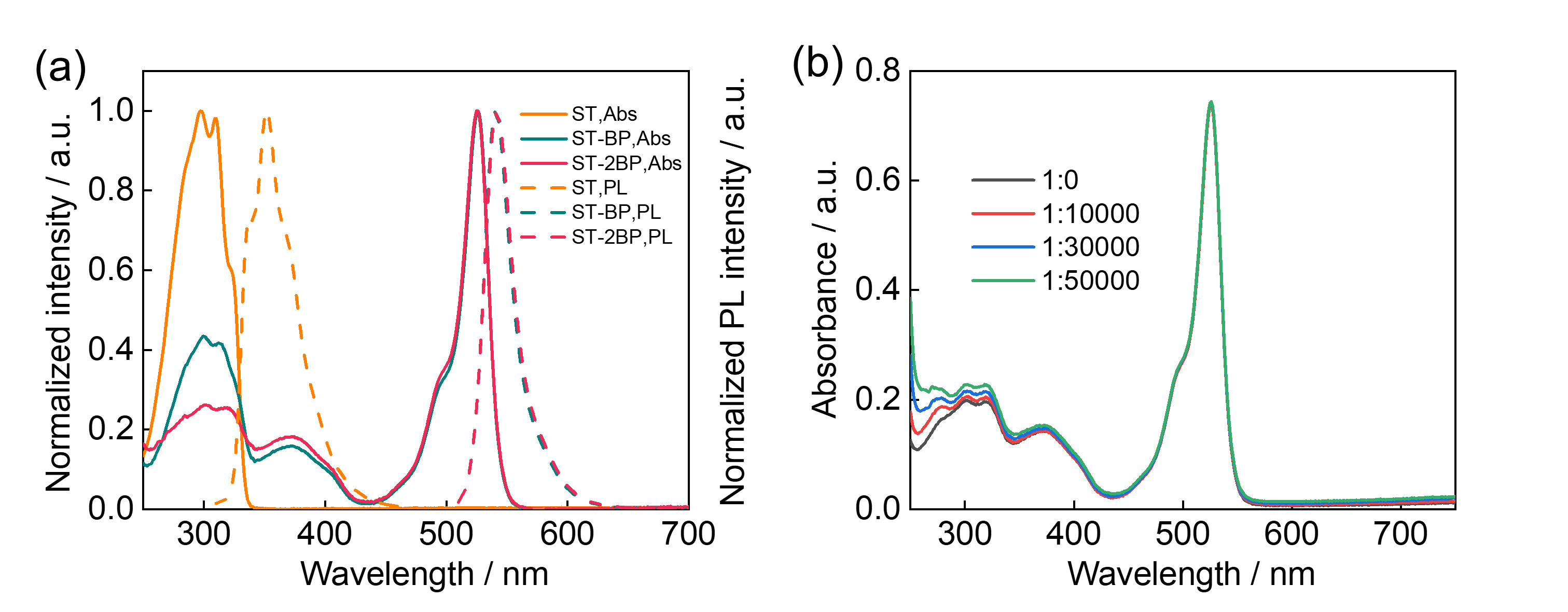


**Figure S7.** (a) UV-vis absorption and fluorescence spectra of ST, **ST-BP**, and **ST-2BP**. (b) UV–vis absorption spectra of **ST-2BP** in DCM solution (10^−6^ M) in the presence of n-hexylamine with ratios of **ST-2BP**:n-hexylamine = 1:0, 1:10000, 1:30000, and 1:50000 mol mol^−1^.


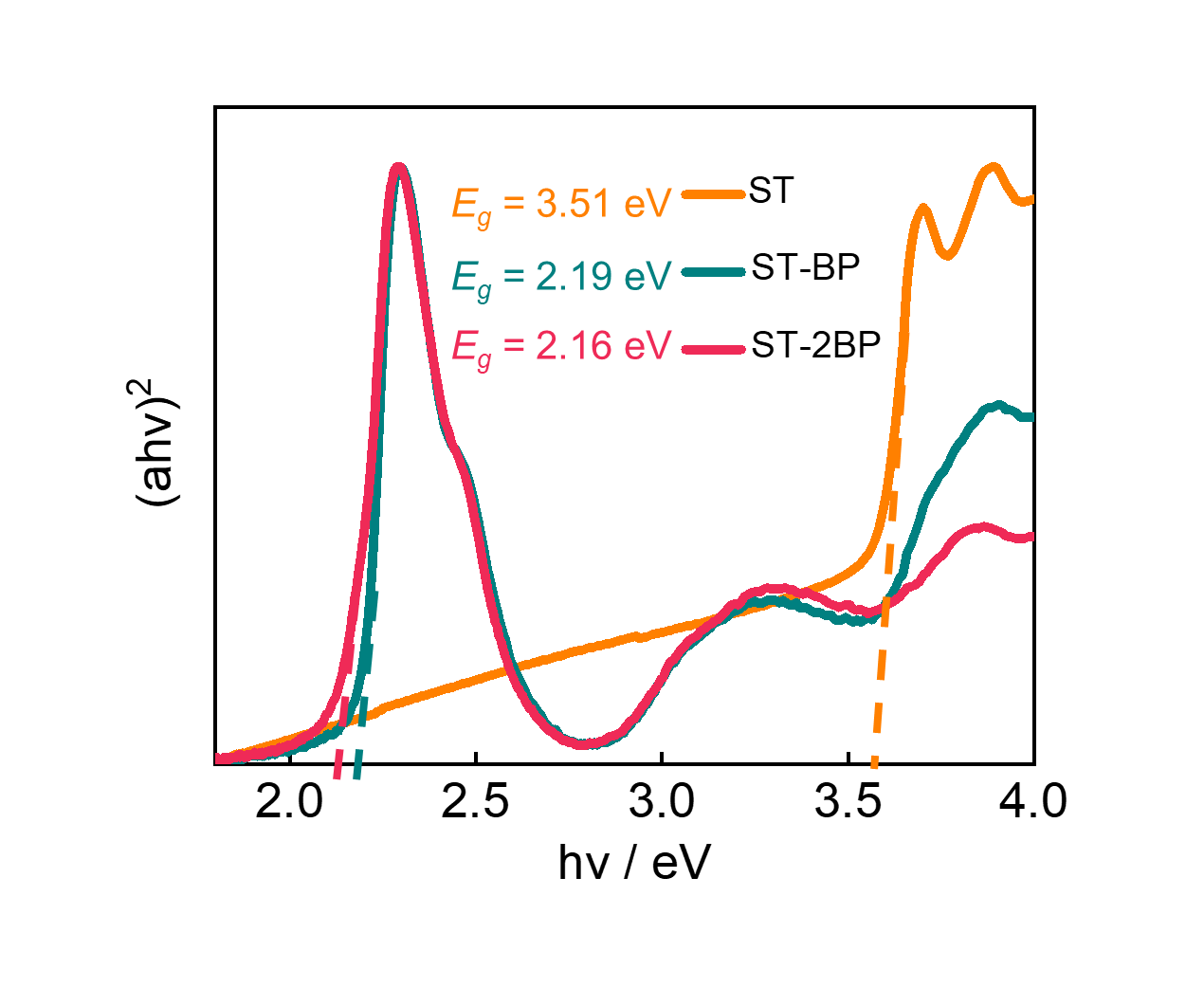


**Figure S8.** Tauc plots of ST, **ST-BP**, and **ST-2BP.**


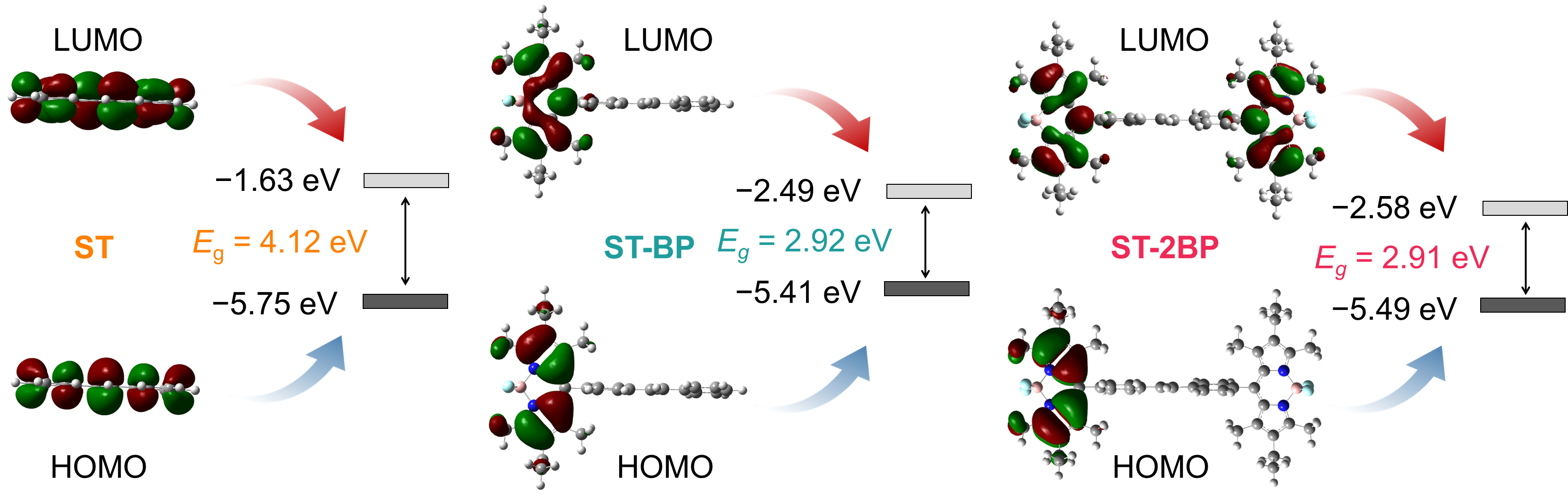


**Figure S9.** Kohn–Sham LUMOs and HOMOs of the model compounds based on the calculations at B3LYP/6-31G(d,p) level, and corresponding LUMO-HOMO levels and bandgaps.


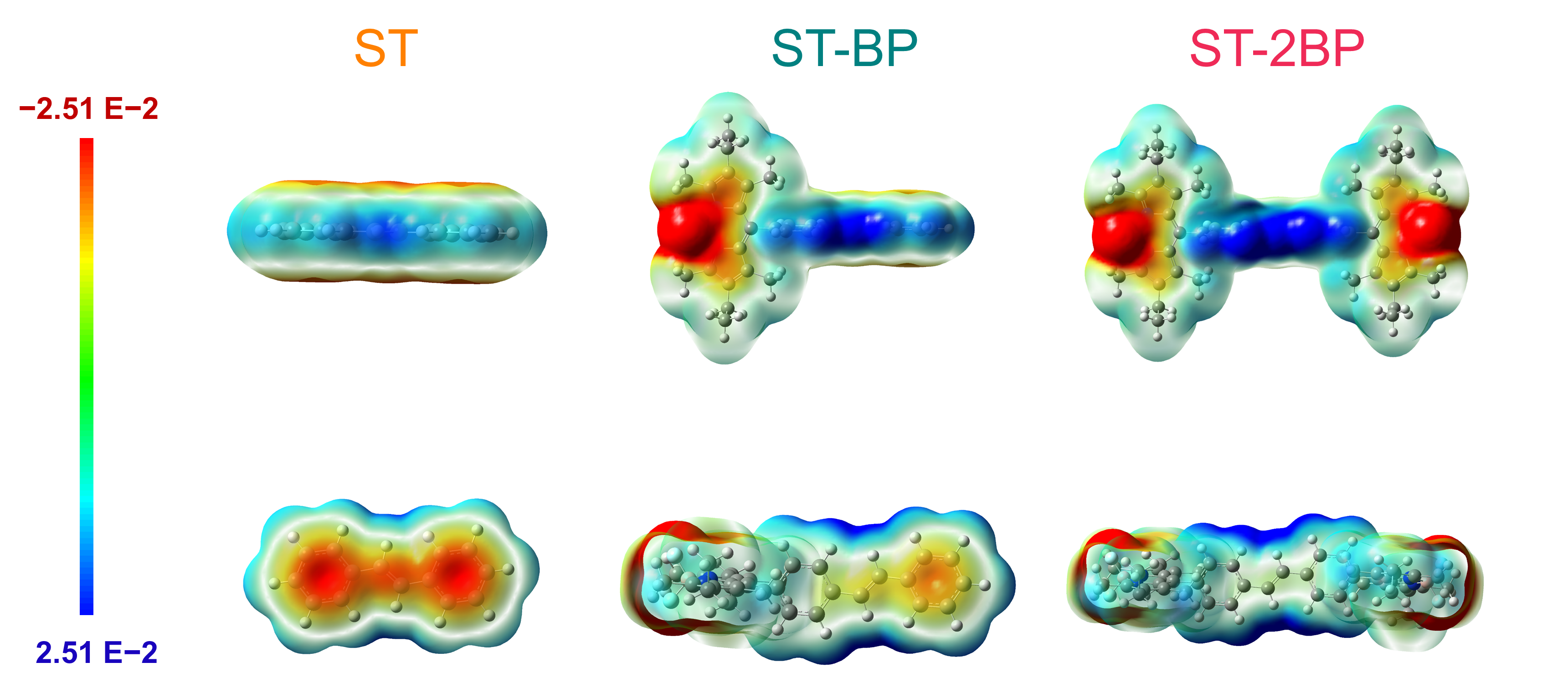


**Figure S10.** The electrostatic potential surface maps of ST, **ST-BP**, and **ST-2BP**.


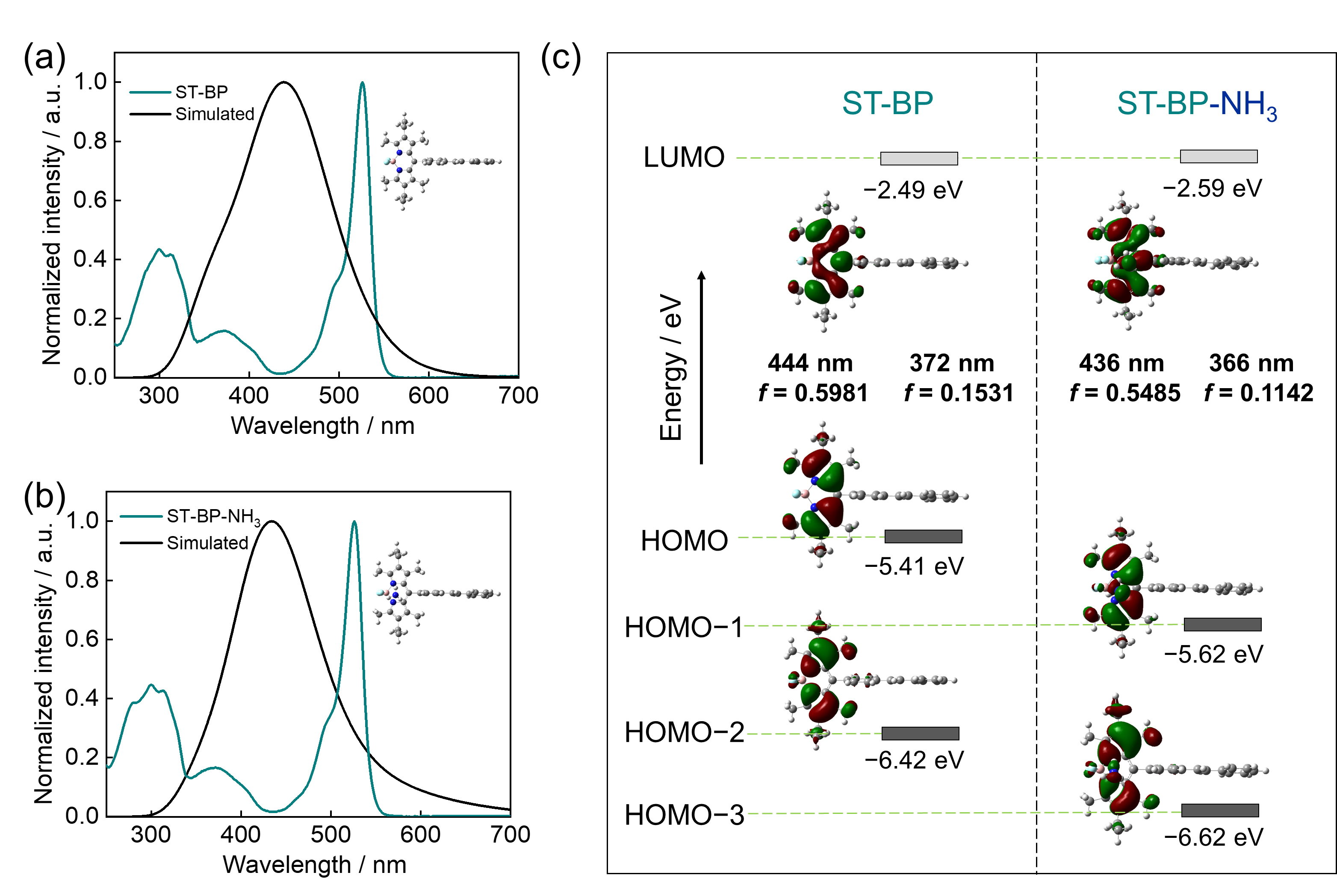


**Figure S11.** UV-vis spectra of (a) **ST-BP** and (b) **ST-BP-NH_3_** obtained through theoretical calculation and testing. (c) Kohn–Sham molecular orbitals of **ST-BP** and **ST-BP-NH_3_**, together with the wavelengths and oscillator strengths for the S_0_→S_3_ and S_0_→S_4_ transitions, based on the DFT calculations.

**Table S1.** Summary of organic small molecules ammonia sensors.


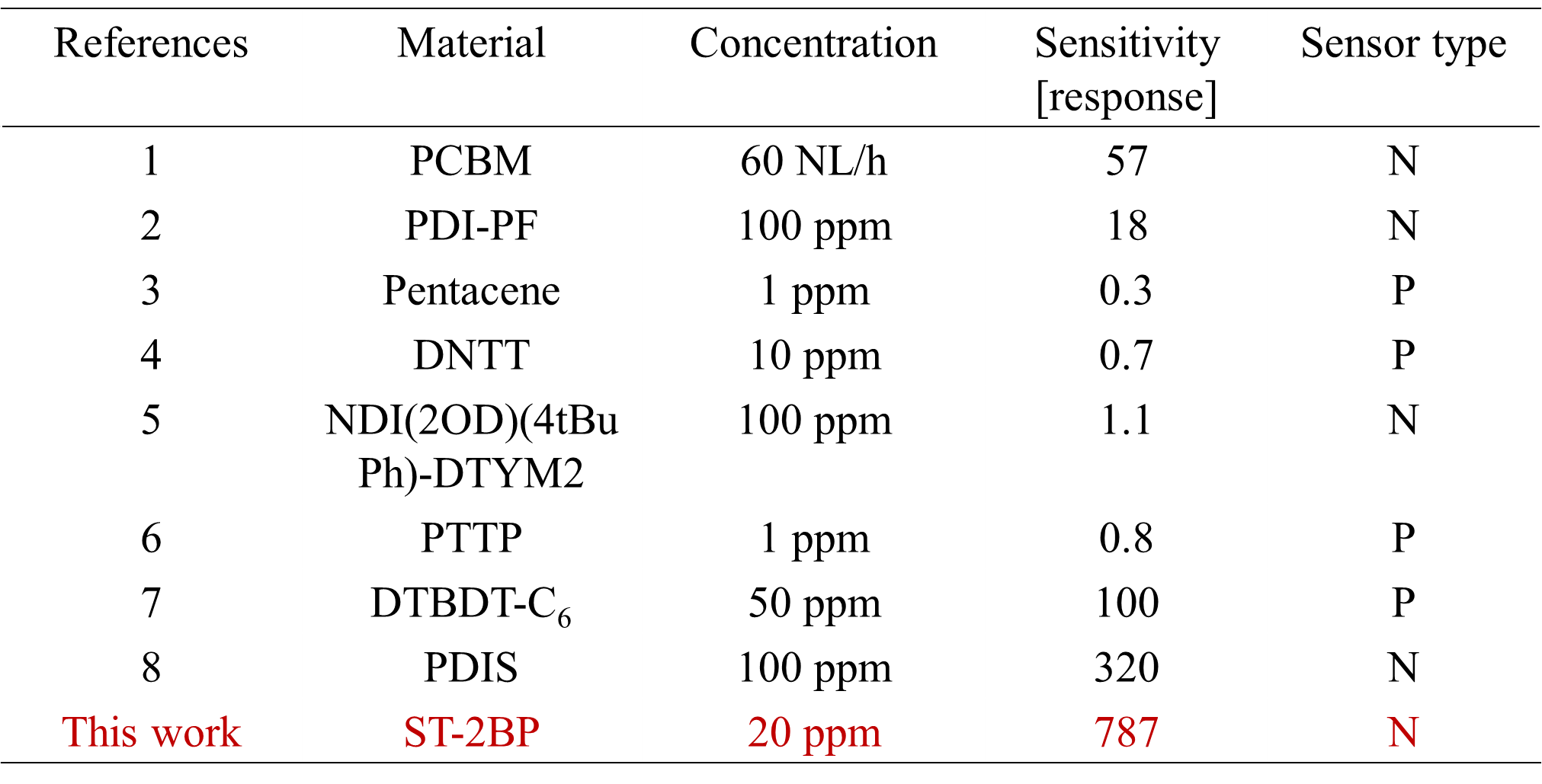


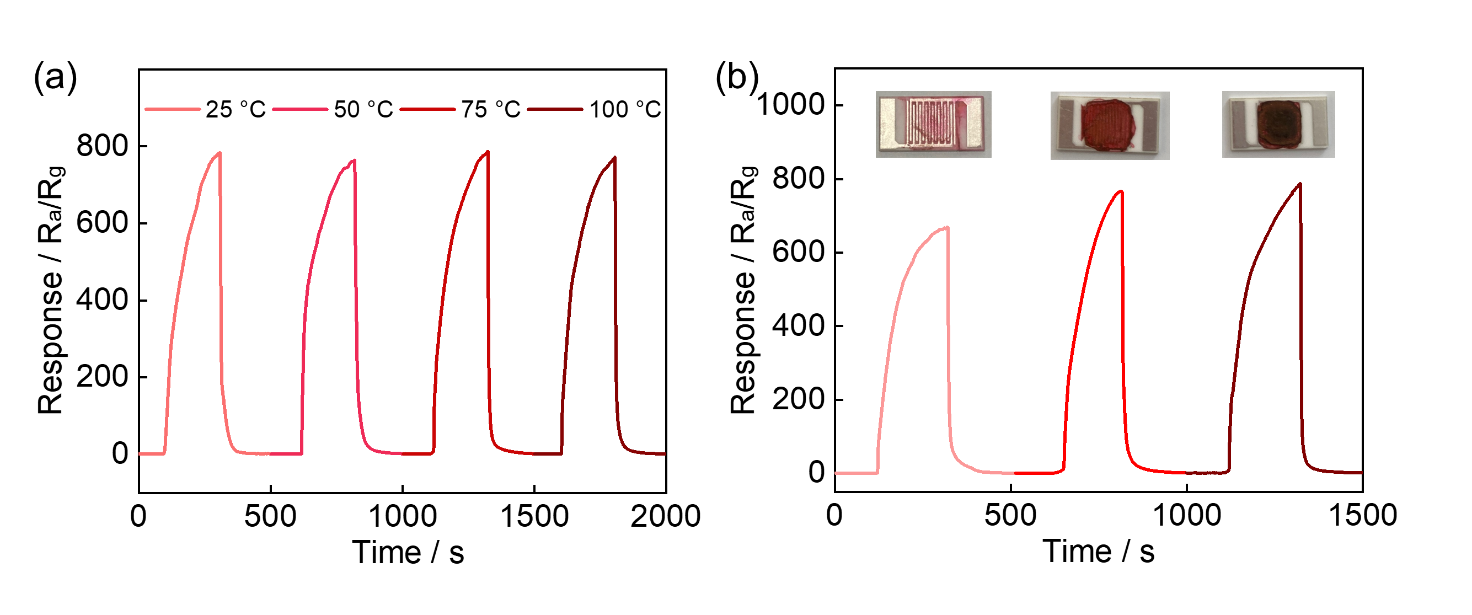


**Figure S12.** (a) Sensing response curves of **ST-2BP** for 20 ppm NH_3_ at different temperatures. (b) Sensing response curves of **ST-2BP** sensing devices loaded with different thicknesses at room temperature under 20 ppm NH_3_.


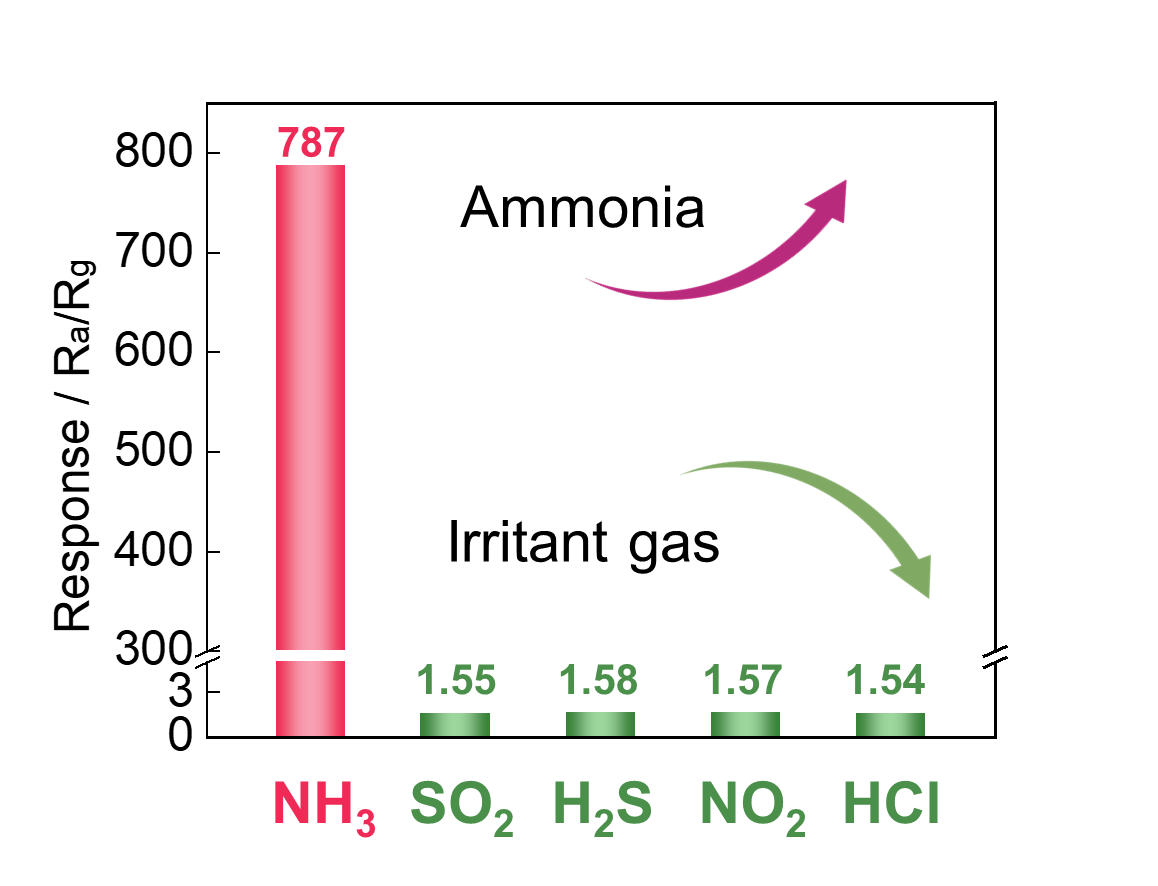


**Figure S13.** Sensing response values of **ST-2BP** in ammonia and four other irritating gases.


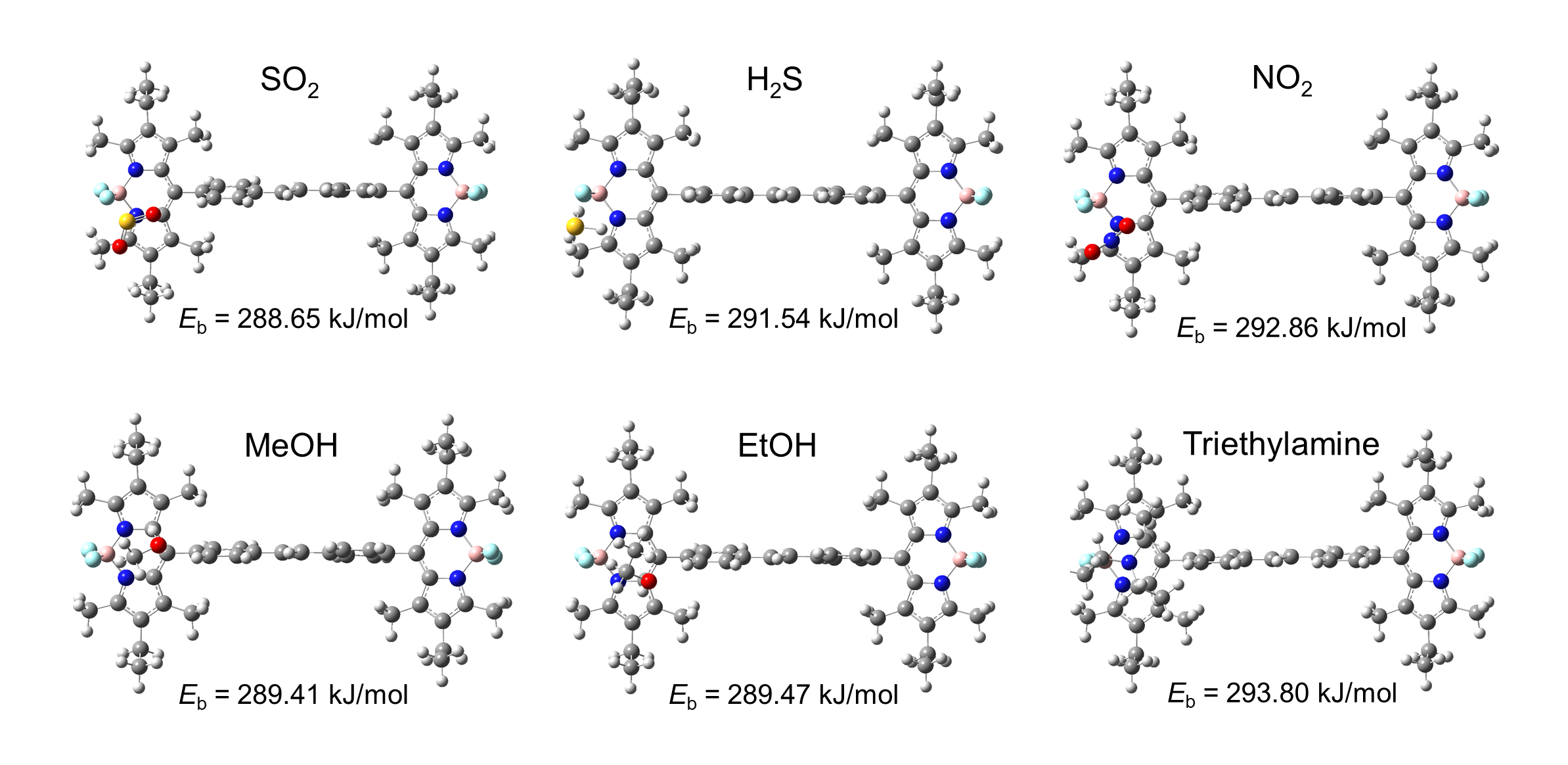


**Figure S14.** The adsorption models of **ST-2BP** and six different gases.


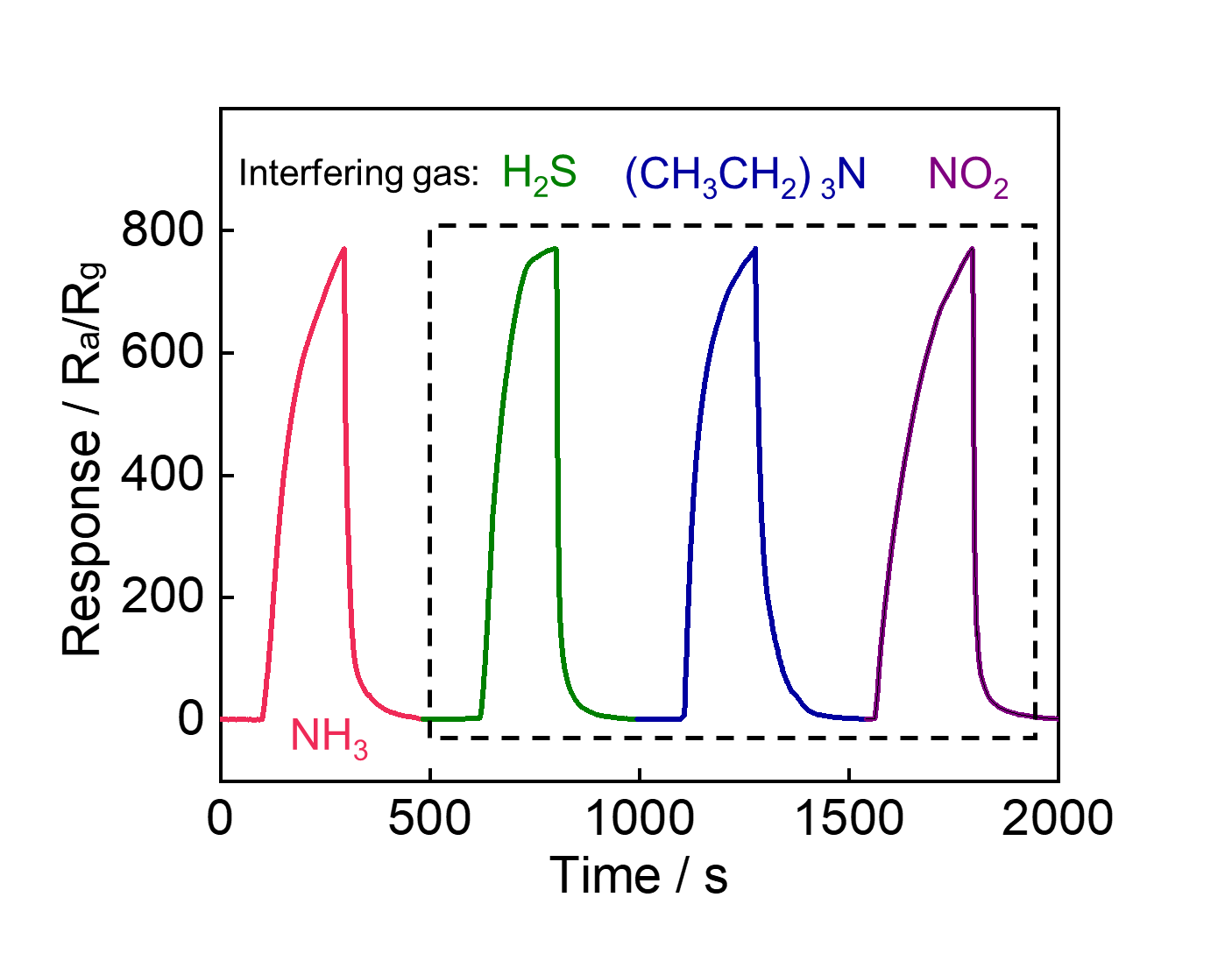


**Figure S15.** The anti-interference performance of the **ST-2BP** sensor in the mixture of ammonia and interfering gas (H_2_S, TEA, and NO_2_).


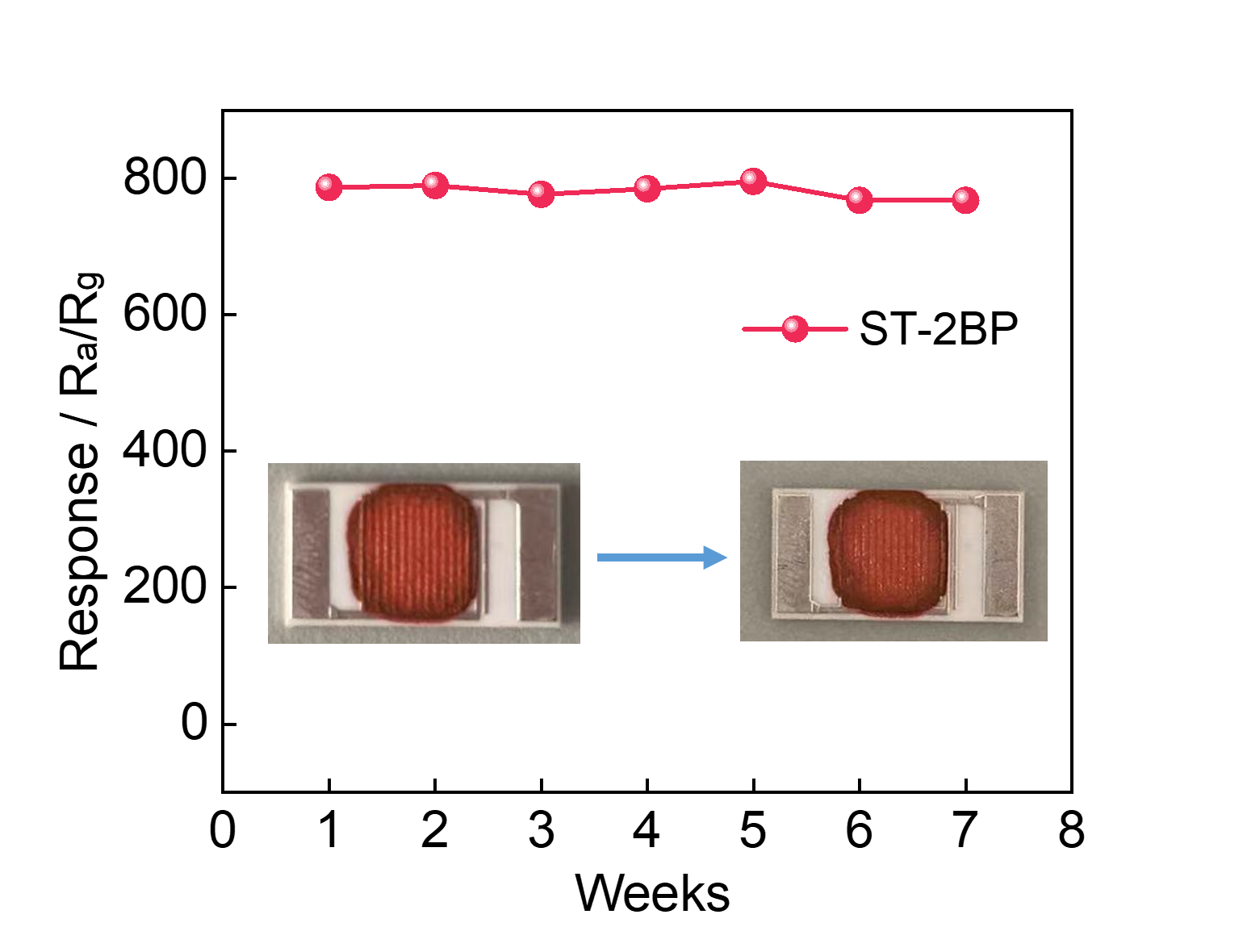


**Figure S16.** Long-term stability of the **ST-2BP** sensor to 20 ppm NH_3_ at room temperature. Insets: optical photos before and after testing.


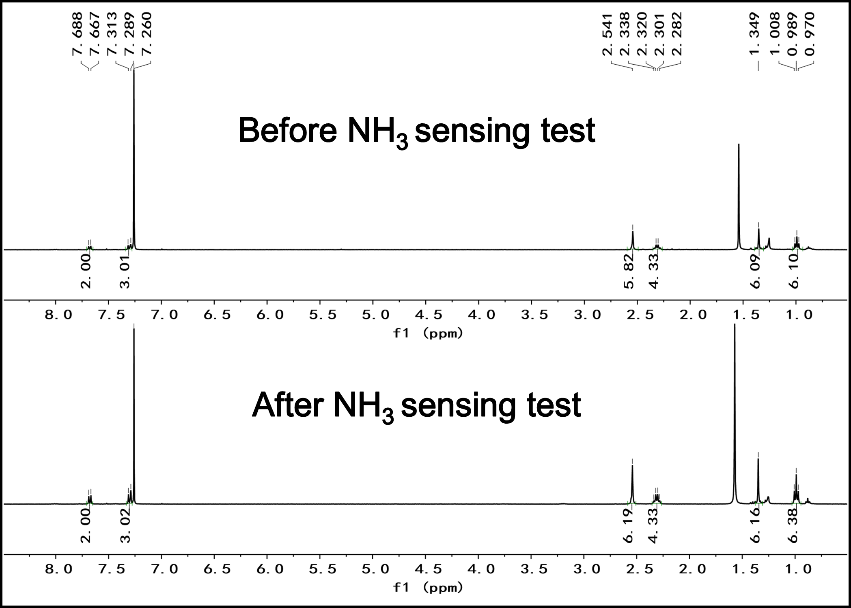


**Figure S17.** The ^1^H NMR comparison of **ST-2BP** before and after sensing testing.


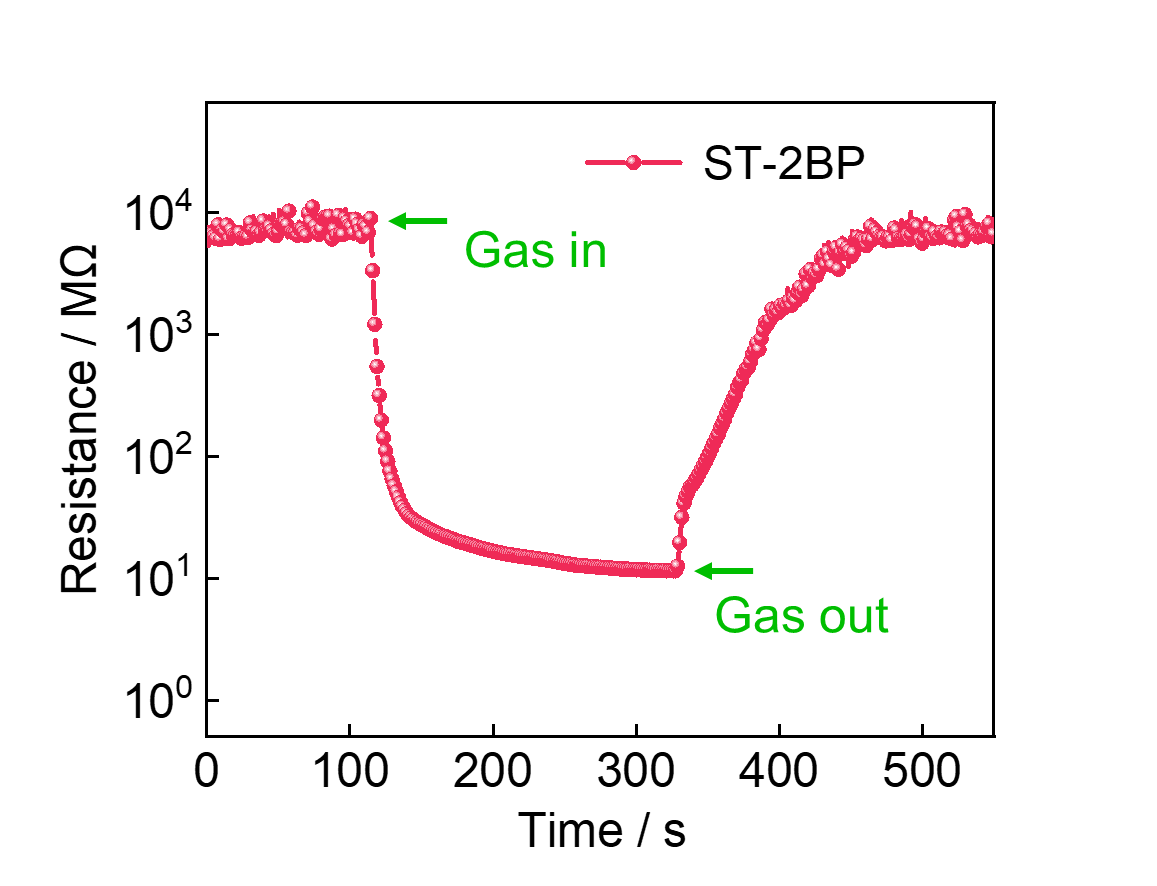


**Figure S18.** The resistance response/recovery curve of the **ST-2BP** to 20 ppm of NH_3_.


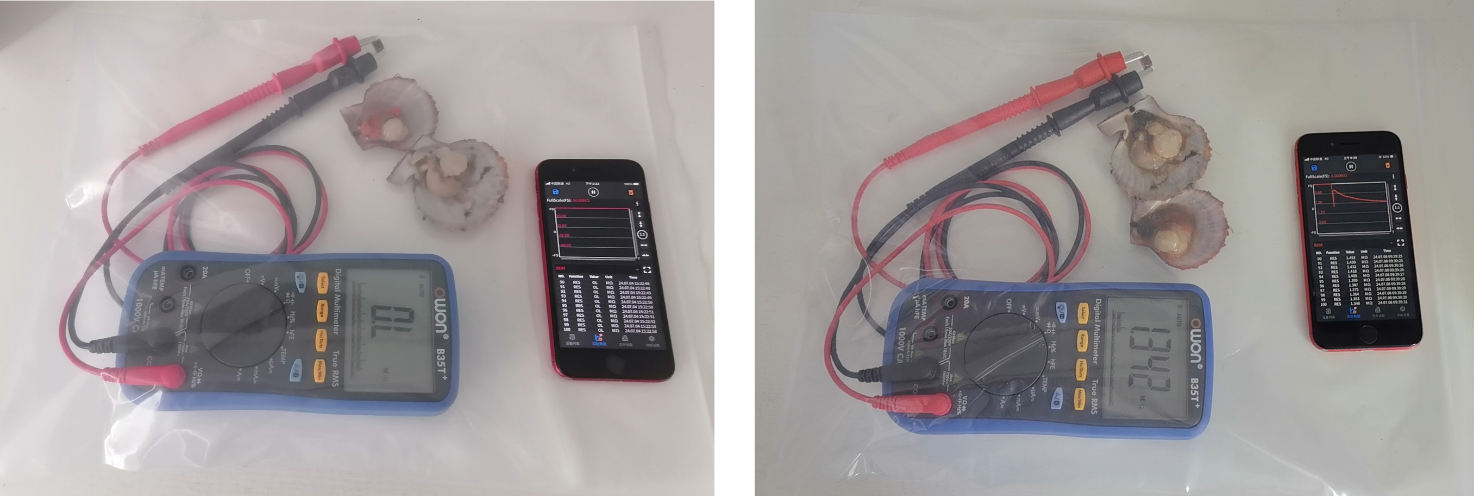


**Figure S19.** Real time resistance of **ST-2BP** sensor before (left) and after (right) seafood spoilage.

**Figure S20.** ^1^H NMR spectrum of **ST-2BP**.

**Figure S21.** ^1^H NMR spectrum of **ST-BP**.

**Figure S22.** ^1^H NMR spectrum of ST.

**Figure S23.** ^1^H NMR spectrum of BP.

**Figure S24.** ^11^B NMR spectrum of **ST-2BP**.

**Figure S25.** ^11^B NMR spectrum of **ST-BP**.

**Figure S26.** ^13^C NMR spectrum of **ST-2BP**.

**Figure S27.** ^13^C NMR spectrum of **ST-BP**.

Supplementary Reference

[1] A. V. S. Simõis, L. K. M. Roncaselli, V. J. R. d. Oliveira, M. E. R. S. Medina, H. H. Ramanitra, M. Stephen, D. L. S. Agostini, R. C. Hiorns, C. d. A. Olivati, *Materials Research.* **2021**, *24*, e20210435.

[2] B. M. Oh, S.-H. Park, J. H. Lee, J. C. Kim, J. B. Lee, H. J. Eun, Y.-S. Lee, B. E. Seo, W. Yoon, J. E. Kwon, H. Yun, S. K. Kwak, O. Kwon, J. H. Kim, *Adv. Funct. Mater.* **2021**, *31*, 2101981.

[3] H.-W. Zan, W.-W. Tsai, Y. Lo, Y.-M. Wu, Y.-S. Yang, *IEEE Sens. J.* **2012**, *12*, 594–601.

[4] J. Lu, D. Liu, J. Zhou, Y. Chu, Y. Chen, X. Wu, J. Huang, *Adv. Funct. Mater.* **2017**, *27*, 1700018.

[5] F. Zhang, C. Di, N. Berdunov, Y. Hu, Y. Hu, X. Gao, Q. Meng, H. Sirringhaus, D. Zhu, *Adv. Mater.* **2013**, *25*, 1401–1407.

[6] D. Liu, Y. Chu, X. Wu, J. Huang, *Sci. China Mater.* **2017**, *60*, 977–984.

[7] L. Li, P. Gao, M. Baumgarten, K. Müllen, N. Lu, H. Fuchs, L. Chi, *Adv. Mater.* **2013**, *25*, 3419–3425.

[8] Q. Deng, E. Zhou, Y. Huang, W. Qing, H. Zhai, Z. Liu, Z. Wei, *Chem. Commun.* **2019**, *55*, 4379.
